# Classifying Integrated Signature Molecules in Macrophages of Rheumatoid Arthritis, Osteoarthritis, and Periodontal Disease: An Omics-Based Study

**DOI:** 10.3390/cimb44080241

**Published:** 2022-08-06

**Authors:** Prachi Sao, Yamini Chand, Lamya Ahmed Al-Keridis, Mohd Saeed, Nawaf Alshammari, Sachidanand Singh

**Affiliations:** 1 Faculty of Biotechnology, Institute of Biosciences and Technology, Shri Ramswaroop Memorial University, Barabanki 225003, Uttar Pradesh, India; 2 Department of Biology, College of Science, Princess Nourah Bint Abdulrahman University, Riyadh 11671, Saudi Arabia; 3 Department of Biology, College of Science, University of Hail, Hail 55476, Saudi Arabia; 4 Department of Biotechnology, Vignan’s Foundation for Science, Technology, and Research (Deemed to be University), Vadlamudi, Guntur 522213, Andhra Pradesh, India; 5 Department of Biotechnology, Smt. S. S. Patel Nootan Science & Commerce College, Sankalchand Patel University, Visnagar 384315, Gujarat, India

**Keywords:** rheumatoid arthritis (RA), osteoarthritis (OA), periodontitis, *Porphyromonas gingivalis* (PG), network biology, gene expression analysis

## Abstract

Rheumatoid arthritis (RA), osteoarthritis (OA), and periodontal disease (PD) are chronic inflammatory diseases that are globally prevalent, and pose a public health concern. The search for a potential mechanism linking PD to RA and OA continues, as it could play a significant role in disease prevention and treatment. Recent studies have linked RA, OA, and PD to *Porphyromonas gingivalis* (PG), a periodontal bacterium, through a similar dysregulation in an inflammatory mechanism. This study aimed to identify potential gene signatures that could assist in early diagnosis as well as gain insight into the molecular mechanisms of these diseases. The expression data sets with the series IDs GSE97779, GSE123492, and GSE24897 for macrophages of RA, OA synovium, and PG stimulated macrophages (PG-SM), respectively, were retrieved and screened for differentially expressed genes (DEGs). The 72 common DEGs among RA, OA, and PG-SM were further subjected to gene–gene correlation analysis. A GeneMANIA interaction network of the 47 highly correlated DEGs comprises 53 nodes and 271 edges. Network centrality analysis identified 15 hub genes, 6 of which are DEGs (*API5*, *ATE1*, *CCNG1*, *EHD1*, *RIN2*, and *STK39*). Additionally, two significantly up-regulated non-hub genes (*IER3* and *RGS16*) showed interactions with hub genes. Functional enrichment analysis of the genes showed that “apoptotic regulation” and “inflammasomes” were among the major pathways. These eight genes can serve as important signatures/targets, and provide new insights into the molecular mechanism of PG-induced RA, OA, and PD.

## 1. Introduction

Arthritis is a prevalent disease. It is often defined as joint swelling, pain, and stiffness. It is a collective term that encompasses a variety of joint inflammation and bone disease conditions. The most frequent kinds of arthritis are rheumatoid arthritis (RA) and osteoarthritis (OA). RA is an autoimmune and inflammatory disease in which the body’s immune system attacks its tissue, including the linings of the joints and internal organs (in extreme cases), resulting in painful swelling and ultimately leading to bone deterioration and disaggregation over time [1,2]. In contrast, OA, the most prevalent kind of arthritis, causes pain, stiffness, joint degeneration, and osteophytes. The majority of research connects OA to low-grade inflammation and mechanical stress, including accidents, aging, obesity, etc. Recent research, however, indicates that different kinds of inflammation may play a key role in the emergence of OA [3,4,5,6]. A study [7] found a correlation between the severity of OA and rising levels of systemic inflammatory markers, such as lipopolysaccharides (LPS) produced by bacteria. Despite having different pathogeneses, both RA and OA share phenotypic traits, cells, and molecular properties [8]. Furthermore, research shows that RA and OA significantly alter the oral microbiome [3]. Periodontal disease (PD) is a chronic inflammatory disease of the tooth and supporting tissues that has been linked to a particular group of bacteria, one of which is *Porphyromonas gingivalis* [9,10,11]. Chronic periodontitis is usually characterized by a heavy infiltrate of inflammatory cells, including macrophages, in the gingival tissue; it results in the resorption of alveolar bone and other tooth-supporting tissues. Epidemiological studies suggest that the involvement of *Porphyromonas gingivalis* (PG) is not just confined to oral diseases, but also a variety of other systemic diseases, such as diabetes, coronary heart disease, Alzheimer’s disease, rheumatoid arthritis, and adverse pregnancy outcomes [9,12,13,14,15,16]. Numerous clinical studies have revealed a possible link between RA and PG; one such study suggests that PG may cause RA by producing anticitrullinated protein/peptide antibodies, which are known markers for RA [3,16,17,18].

According to Han and colleagues [19], oral pathogens can enter the bloodstream and spread throughout the body via moderately porous epithelial pores. When macrophages are challenged with PG, the microtubule-associated protein 1 light chain 3 (LC3) and its intracellular sorting protein partner melanoregulin (MREG) become linked; this relationship obstructs the host immune system’s clearance of the pathogen [19]. Macrophages produce pro-inflammatory cytokines during infection to initiate the host immune response [20]. Also, a lot of research has suggested a significant role of macrophages in the pathogenesis of chronic inflammatory conditions such as PD and RA [21,22]. In general, macrophages are polarized into pro-inflammatory macrophages (M1) and anti-inflammatory macrophages (M2) based on the type of cytokines and phenotypes they produce. In certain studies, disease development has been linked to abnormal M1 and M2 polarization [23,24]. In the oral environment, macrophages adopt an M1 phenotype during the acute phase of inflammation (chronic PD condition) to eradicate invading pathogens and then transition to M2, which increases migration and immunosuppression activity [25]. However, in RA and OA, macrophages drive synovial inflammation through the release of pro-inflammatory cytokines, degradation of the extracellular matrix, and recruitment of other immune cells such as neutrophils [26,27]. Numerous studies have revealed the presence of PG in synovial tissue, where PG, along with IL-1 and TNF-alpha, induce fibroblasts and lead to dysregulation of osteoclasts and osteoblasts; this results in bone resorption [17,28,29,30].

Therefore, investigating the genes that are differentially expressed in macrophages will provide insight into the common pathogenic mechanisms behind these disease states, and since macrophages are the first to come into contact with invading pathogens, they are also the best site to study host-pathogen interactions for the identification of signature molecules for treatment and early diagnosis of RA, OA, and PD. While it is known that these three diseases share overlapping pathophysiological markers and processes, the precise mechanism of initiation and progression is not well understood. Previous studies have focused on finding biomarkers/targets that are specific to either RA, OA, or PD [3,31,32,33], but a biomarker that correlates with all three diseases is still not understood. The investigation of multifactorial diseases has turned toward data sharing, analyzing, and integration of many kinds of data, such as genomics, transcriptomics, and proteomics. Multiple-associated diseases analyzed by integrating different microarray expression profiles have already shown huge success in the identification of biomarkers and targets [15,34]. Incorporating protein-protein interaction networks with the above-mentioned concept helps in explaining different forms of biological processes, and in predicting molecular functions that have already existed in previous research work and textbooks [31,35,36,37]. There are instances in which proteins and genes may not be related directly but interact in the same pathway, or may work in tandem with each other in different phenomena in the same biological processes [38].

In the present study, we retrieved the publicly available gene expression profiles from the Gene Expression Omnibus (GEO) database containing samples for macrophages in rheumatoid arthritis (RA) synovium (GSE97779), osteoarthritis (OA) synovium (GSE123492), and *Porphyromonas gingivalis* stimulated macrophages (PG-SM) (GSE24897). All three data sets were screened for differentially expressed genes (DEGs) using R. The common DEGs among RA, OA, and PG-SM were selected and subjected to gene–gene correlation analysis. Additionally, an interaction network for the highly correlated DEGs was built using GeneMANIA, and the cytoHubba plugin for Cystoscope was used to calculate network centrality metrics, in order to identify important nodes in the network. Finally, functional enrichment analysis was performed for highly interacting (hub) genes using Enrichr and DAVID. The identification of hub genes shared among RA, OA, and PD, in addition to the analysis of their biological processes and pathways, may shed light on the molecular mechanism of the pathogenesis of these diseases.

## 2. Materials and Methods

**Figure 1:**
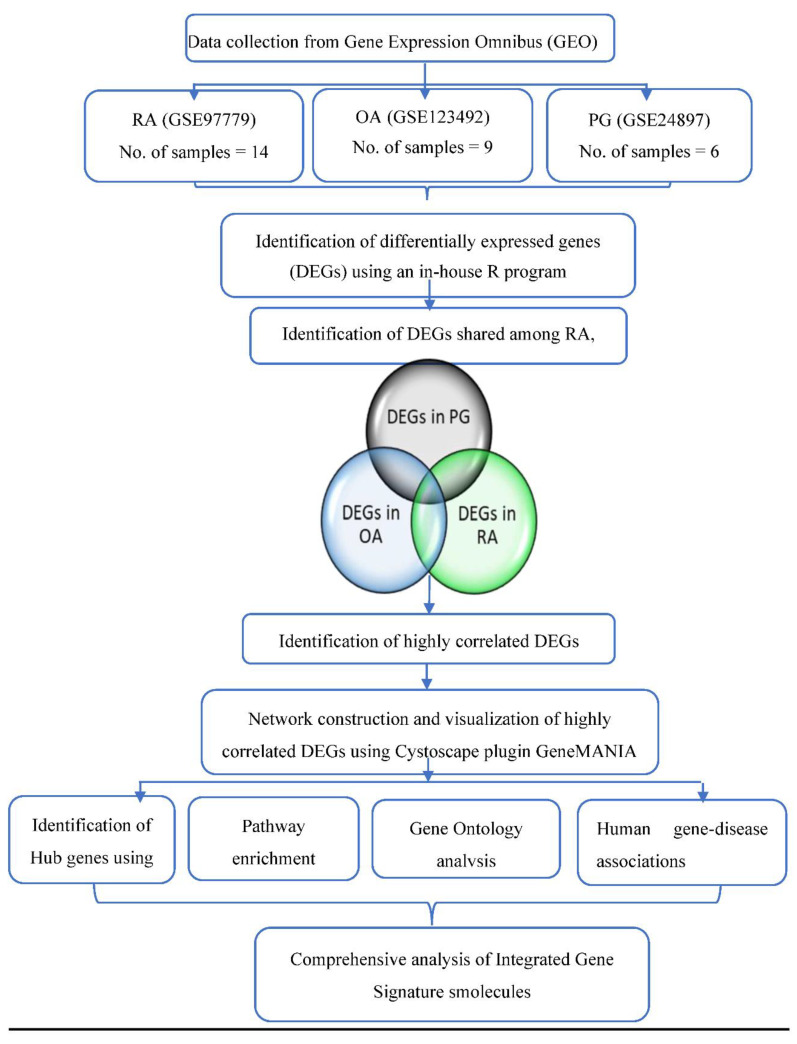
Workflow of the present study. GEO = Gene Expression Omnibus, RA = rheumatoid arthritis, OA = osteoarthritis, PG = *Porphyromonas gingivalis*, DEGs = differentially expressed genes.

In the present study, we analyzed the gene expression profiles of macrophages in RA synovium, OA synovium, and PG-SM, in order to identify common significant biomarkers/targets associated with the three conditions. Figure 1 depicts the workflow of the study.

### 2.1. Retrieval of Gene Expression Data

The gene expression data were retrieved from the Gene Expression Omnibus (GEO) database (https://www.ncbi.nlm.nih.gov/geo/) (date accessed: 6 September 2021) [34] using the following search terms: *Porphyromonas gingivalis*, rheumatoid arthritis, and osteoarthritis, as well as the following filters: organism (*Homo sapiens*) and tissue type (macrophages). Two data sets, GSE97779 and GSE24897, for macrophages in RA and PG-SM, respectively, were selected. A third RNAseq data set for OA, series ID GSE123492, was also selected to examine the DEGs at the transcriptional level. The details of the data sets are listed in Table 1. The data sets GSE97779 and GSE24897 were generated using the Affymetrix Human Genome U133 Plus 2.0 Array platform. GSE97779 contains samples from RA synovial macrophages (nine) and control macrophages (five) [39], whereas GSE24897 contains twelve samples from four conditions: macrophages treated with saline (three), PG (three), PG-LPS (lipopolysaccharide) (three), and PG-fimbriae (three) [6]. This data set was incorporated in the study to examine the effect of PG virulence factors on adherent macrophages, in order to gain a better understanding of the intricate relationships between innate immune response in human macrophages and chronic PD. Of the total twelve samples, six sample macrophages treated with PG and saline were selected for the present analysis. The data set GSE123492 was constructed with the Illumina NextSeq 500 platform, and contains samples from synovial tissue macrophages in OA (nine), RA (two), and PD (one) [20]. OA data were subgrouped as classic OA (cOA; five) and inflammatory OA (iOA; four). For our analysis, only nine OA samples were used. 

### 2.2. Identification of Differentially Expressed Genes (DEGs)

The GEOquery package of R software [40] was used to read the Series Matrix File(s) of the data sets GSE97779 and GSE24897, and a log2 transformation was applied to make them more symmetrical so that the parametric statistical test would produce more accurate and meaningful differentially expressed genes (DEGs). Two categories of probes have been excluded to eliminate false-positive statistical findings: (1) Affymetrix controls with the symbol “AFFX” and (2) probe sets that are likely to hybridize with several genes (Affymetrix designates these probe sets with the abbreviation x). DEGs were screened using the Linear Models for Microarray Analysis (Limma; www.bioconductor.org/packages/release/bioc/html/limma.html) package in R (date accessed: 6 September 2021) [41,42]. A *t*-test in the Limma package was performed to determine the *p*-value of each gene symbol. The following criteria were used to identify significant DEGs: *p*-value ≤ 0.05 and log2-fold change (logFC) ≥ ±1 [8]. Gene annotations were retrieved from the GEO database and prob ids were transformed into gene symbols; genes with the lowest *p*-values were chosen in the case of duplicate genes. The Raw Count Matrix Files for GSE123492 were also read using the GEOquery package and were normalized using the EdgeR tool of R [37]. After normalizing the data, DEGs were identified using the Fisher’s Exact Test between cOA and iOA. The following criteria were used to identify significant DEGs: *p*-value ≤ 0.05 and log2-fold change (logFC) ≥ ±1. Finally, a gene set of common DEGs were identified in RA, OA, and PG-SM.

### 2.3. Gene–Gene Correlation Network Construction

A correlation network was built for the common DEGs based on their expressions. Expression data for 72 common DEGs for each RA, OA, and PG-SM were extracted. Next, individual gene interactions were determined using Pearson correlation coefficients between pairs of 72 common DEGs independently for RA, OA, and PG-SM. Three co-expression-based matrices between each of the 72 common DEGs were created using the Cladist tool [31,42]. Cladist takes expression values as input and computes an *N* × *N* co-expression matrix between each gene pair by employing the Pearson correlation coefficient (r) score given by the following:

(1) $$r=\frac{\sum_{i=1}^{n}\left(x_{i}-\bar{x}\right)\left(y_{i}-\bar{y}\right)}{\sqrt{\sum_{i=1}^{n}{\left(x_{i}-\bar{x}\right)}^{2}\sum_{i=1}^{n}{\left(y_{i}-\bar{y}\right)}^{2}}}$$

where $\bar{x}$ and $\bar{y}$ represent the sample means of the expression values of the two genes in the control and diseased state, respectively. The r score ranges from −1 to +1; the higher the r score, the greater the expression similarity between two genes.

Extensive gene–gene correlation analysis produced a significantly large amount of data; therefore, non-repeating highly correlated gene–gene pairs with an r score > 0.7 were extracted individually for all three disease conditions [43]. 

### 2.4. Network Construction and Hub Gene Identification

#### 2.4.1. Network Construction 

The Cytoscape [44] plugin GeneMANIA [45] v3.5.2 (https://apps.cytoscape.org/apps/genemania) (date accessed: 6 September 2021) was used to construct an interaction network of the DEGs. Cytoscape is an open-source platform-independent network visualization software. It offers several plugins/apps for various network analyses. GeneMANIA allows users to construct a weighted composite gene–gene functional interaction network from a gene list. Functional interactions between the 47 highly correlated common DEGs were predicted by GeneMANIA. In addition to the DEGs, 20 additional genes were used to create the interaction network using *Homo sapiens* as a source species. The functional associations in the network were evaluated using the following terms: co-expression, co-localization, genetic interactions, pathways, physical interactions, predicted interactions, and shared protein domains.

#### 2.4.2. Hub Genes Identification 

Networks can be used to display a wide range of biological data, including protein–protein interactions, gene regulation, cellular pathways, and signal transductions [10,46]. An interaction network is represented as a graph G=(V, E), where V  and E are the sets of vertices (nodes/genes/proteins) and edges (links/functional associations/interactions), respectively [47]. Most biological networks have a scale-free topology and therefore are more robust than random networks. Scale-free networks have a power-law degree distribution, with a small number of highly connected nodes (“hubs”) and a large number of poorly connected nodes (“non-hubs”). Hubs play a significant role in the functional and modular architecture of interactomes. As a result, they are assumed to be more vital to life than non-hub nodes, according to the centrality-lethality rule [48] 

The Cytoscape plugin cytoHubba [49] (https://apps.cytoscape.org/apps/cytohubba) (date accessed: 6 September 2021) was used to calculate the topological parameters of the network. CytoHubba offers 11 topological analysis methods, including six centrality measures. We selected degree centrality (DC), betweenness centrality (BC), bottleneck (BN), and closeness centrality (CC), in order to identify key/important nodes in the network. DC of a node u∊V(G) is defined as the number of its first neighbors. Nodes with high degrees are referred to as “hubs” [47]. BC is a measure of the number of non-redundant shortest paths that pass through a given node. Nodes with high BC are defined as “bottlenecks”, as these nodes act as bridges/connecting links between dense clusters; they control the information flow among other nodes in the network. The BC of a node u∊V(G)  is computed as follows:

(2) $$BC\left(u\right)=\sum_{s\neq t\neq u∊V}\left[\frac{\sigma_{st}\left(u\right)}{\sigma_{st}}\right]$$

where $\sigma_{st}\;$ is the total number of shortest paths from node ‘*s*’ to node ‘*t*’, and $\sigma_{st}\left(u\right)$ is the number of those paths passing through u [47]. Both hubs and bottlenecks tend to be essential in protein interaction networks [48,50].

*CC* is defined as a measure of how fast the flow of information would be from a given node to all other nodes in a network, sequentially. Nodes with high *CC* are the closest to all other nodes in a network. The CC of a node u∊V(G)  is computed as follows:

(3) $$CC\left(u\right)=\frac{N-1}{\sum_{v∊N}d\left(u,v\right)}$$

where $d_{uv}$ is the distance (length of the shortest path) between nodes u and v, and N is the number of nodes in G [47].

### 2.5. Gene Enrichment Analysis

In order to analyze the role of hub genes in RA, OA, and PG-SM, a functional enrichment analysis was performed [37] using two web servers, namely Enrichr (https://maayanlab.cloud/Enrichr/) (date accessed: 6 September 2021) [51] and DAVID v6.8 (Database for Annotation, Visualization, and Integrated Discovery database) (david.ncifcrf.gov/) (date accessed: 6 September 2021) [52]. Enrichr was first used to extract gene ontology (GO) annotations such as biological process (BP), molecular function (MF), and cellular component (CC). Enrichr was then used to perform KEGG (Kyoto Encyclopedia of Genes and Genomes) pathway analysis, which was further confirmed using DAVID. Finally, an Enrichr feature called “Jensen DISEASES” was used to integrate evidence on disease–gene associations [53].

## 3. Results

### 3.1. Identification of DEGs

**Figure 2:**
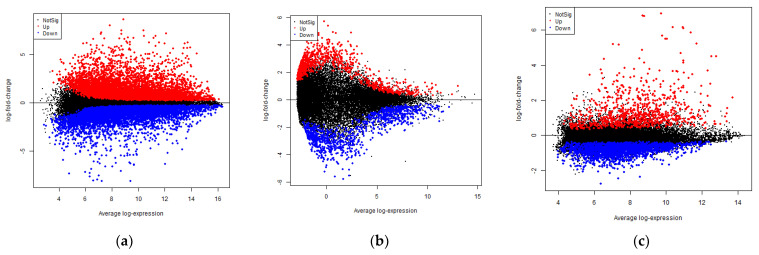
MA plots illustrating the distribution of differentially expressed genes (DEGs) in (**a**) rheumatoid arthritis (RA), (**b**) osteoarthritis (OA), and (**c**) *Porphyromonas gingivalis* (PG) macrophages based on the following statistical parameters: *p*-value ≤ 0.05 and log2-fold change (logFC) ≥ ±1. The red and blue dots indicate genes that have been considerably up- and down-regulated, respectively, whereas the black dots indicate genes with no significant difference between the diseased and control groups.

**Figure 3:**
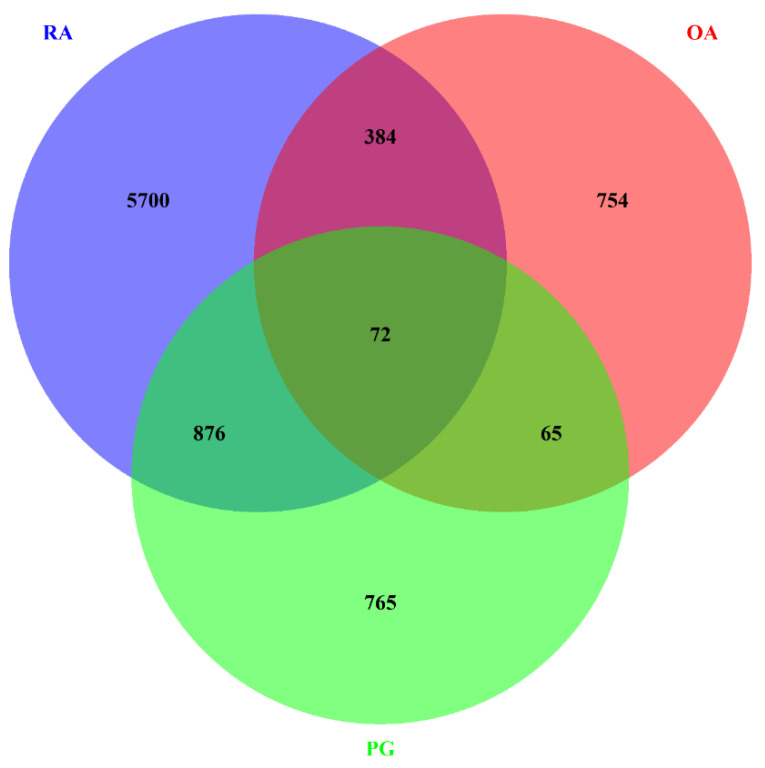
Venn diagram representing the intersection of differentially expressed genes (DEGs) in rheumatoid arthritis (RA), osteoarthritis (OA), and *Porphyromonas gingivalis* (PG) macrophages. All three data sets shared 72 DEGs.

The data sets with the series ID GSE97779, GSE123492, and GSE24897 were subjected to DEG analysis using R. A total of 10890, 1276, and 2340 DEGs were identified in RA, OA, and PG-SM, respectively, based on the following statistical parameters: *p*-value ≤ 0.05 and log2-fold change (logFC) ≥ ±1. An MA-plot (M, log-ratio; A, mean average) for each data set is shown in Figure 2. A total of 72 common DEGs were identified among RA, OA, and PG (Figure 3). 

### 3.2. Gene–Gene Correlation Network Construction

**Figure 4:**
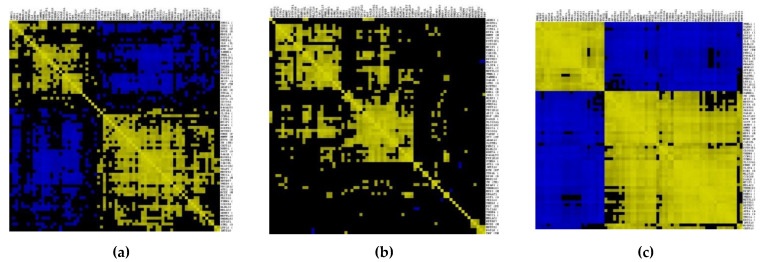
A gene–gene correlation grid map illustrating the correlation between an expression similarity matrix across multiple samples in synovial macrophages in (**a**) rheumatoid arthritis (RA), (**b**) osteoarthritis (OA), and (**c**) *Porphyromonas gingivalis* (PG) stimulated macrophages individually using Cladist. A Pearson correlation coefficient (r) score of >0.7 was used to screen out significantly correlated gene–gene pairs. Pairs of genes that are co-expressed in similar patterns are labeled yellow; anti-correlated genes are designated blue, and those with no relationship are rendered black according to the correlation coefficient threshold.

**Figure 5:**
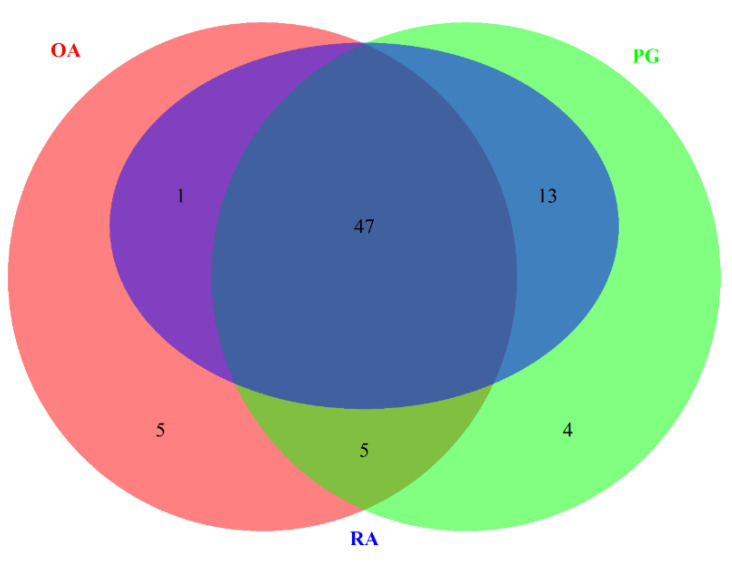

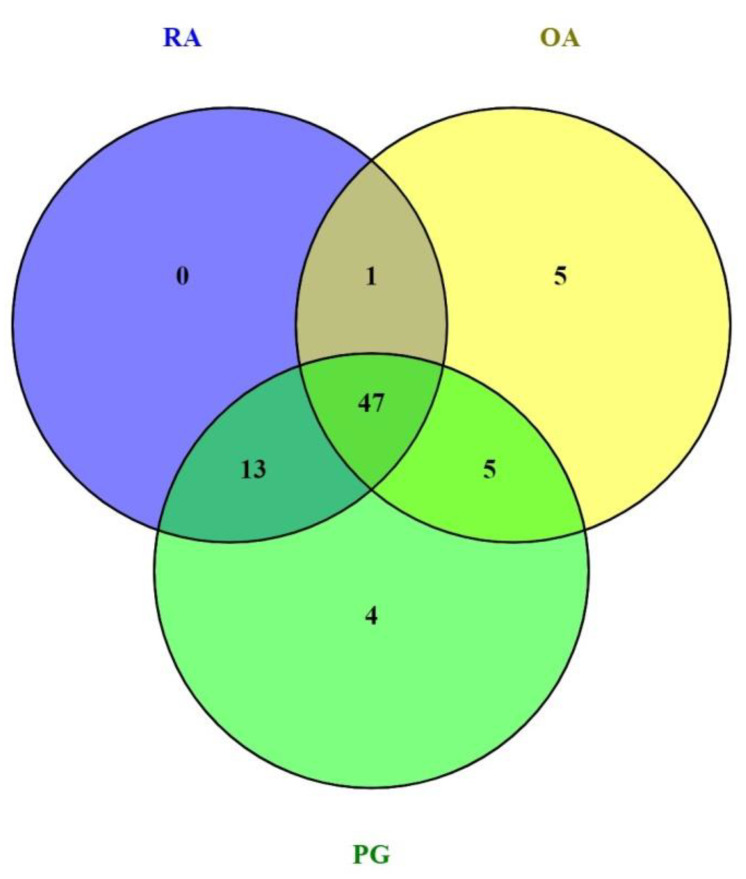
Venn diagram depicting the intersection of significantly correlated differentially expressed genes (DEGs) in macrophages of rheumatoid (RA) synovium, osteoarthritis (OA) synovium, and macrophages stimulated with *Porphyromonas gingivalis* (PG), using a Pearson correlation coefficient (r) score of >0.7. Forty-seven highly correlated DEGs were identified. (why is the blue circle small?).

A total of 72 genes common in RA, OA, and PG-SM were selected for the gene–gene correlation network (Supplementary File: Table S1). The expression values of each sample for the 72 common DEGs in RA, OA, and PG-SM were extracted individually. Three gene–gene correlation matrices were constructed using the Cladist tool [40,43]. Forty-seven genes out of seventy-two common DEGs were highly correlated based on their Pearson correlation coefficient (r-value) values. Table 2 shows the r-values, logFCs, and *p*-values for each of the 47 highly correlated DEGs in RA, OA, and PG-SM. Genes were selected based on a Pearson correlation coefficient (r score) > 0.7. A gene–gene correlation grid map for the overlapping highly correlated DEGs in RA, OA, and PG-SM is shown in Figure 4, and a Venn diagram representing the overlapping highly correlated gene set is shown in Figure 5. The genes were separated into up- and down-regulated genes based on logFC ≥ ±1. Two genes, *IER3* and *RGS16*, were found to be up-regulated in all three data sets; as a result, they were utilized for additional investigation. These genes may be crucial in the shared pathogenic pathways that underlie the states of these disorders. 

### 3.3. Network Construction and Hub Genes Identification

#### 3.3.1. Network Construction 

**Figure 6:**
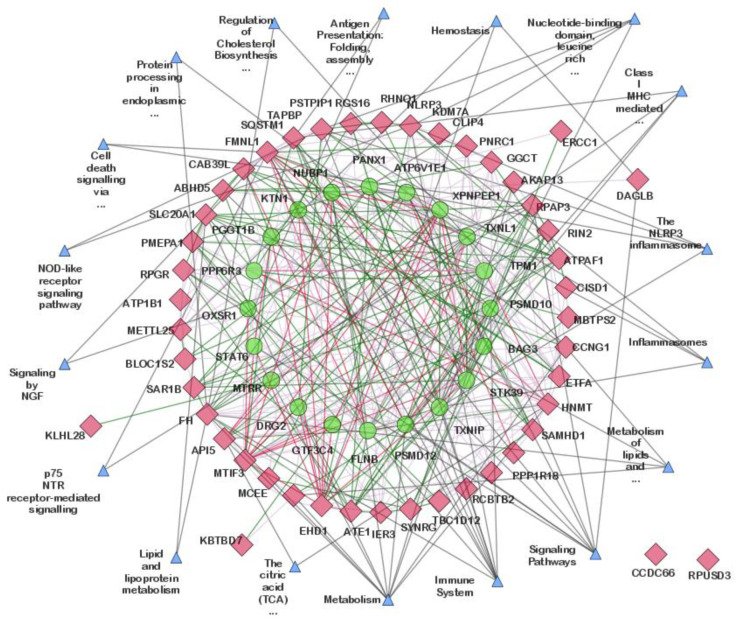
The interaction network of highly correlated common differentially expressed genes (DEGs) as predicted using the GeneMANIA plugin of Cytoscape. The red and green nodes represent common DEGs and related genes, respectively. The red, green, purple, and grey edges represent physical interactions, genetic interactions, co-expression, and consolidated pathways, respectively.

The GeneMANIA plugin was used to predict interactions between the 47 highly correlated common DEGs and 20 additional related genes in the network, using *Homo sapiens* as the source organism. The network comprised 85 nodes and 399 edges (Supplementary File: Table S2). The pathway interaction information from GeneMANIA showed that the majority of the genes were found to be participating in “*Metabolism*”, “*Immune System*”, “*Signaling Pathways*”, “*Class I MHC mediated antigen processing & presentation*”, “*Inflammasomes*”, “*Metabolism of lipids*”, “*Nucleotide-binding domain*, *leucine rich repeat containing receptor (NLR) signaling pathways*”, and “*The NLRP3 inflammasome*”. However, our work was focused on physical and genetic interactions to better understand the physical and molecular mechanisms associated with the hub genes. Therefore, all the nodes with less than two edges were removed, and pathway interactions were omitted, leaving a network with 53 nodes and 271 edges (Figure 6). Among the 53 nodes, 33 were DEGs, and the remaining 20 were related genes.

#### 3.3.2. Identification of Hub Genes

**Figure 7:**
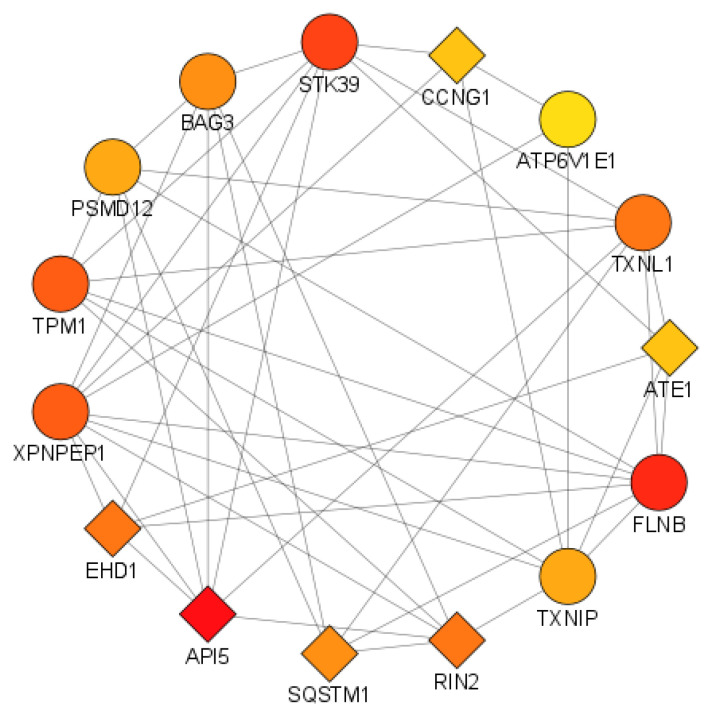
Subnetwork of top 15 hub genes identified by cytoHubba. The intensity of the node’s color denotes the degree of interaction (red = high DC, orange = intermediate DC, and yellow = low DC), whereas the shapes of the nodes represent query/DEG (diamond) and result/related gene (circle).

The Cytoscape plugin cytoHubba was used to calculate the network centrality parameters. In order to identify key nodes in the interaction network, the parameters DC, BC, BN, and CC were selected. The top 15 nodes with high DCs were selected to create a subnetwork of hub genes (Figure 7). Among the top 15 hub genes, 6 genes, namely *API5*, *ATE1*, *CCNG1*, *EHD1*, *RIN2*, and *SQSTM1*, were common DEGs, while the remaining (*ATP6V1E1*, *BAG3*, *FLNB*, *PSMD12*, *STK39*, *TPM1*, *TXNIP*, *TXNL1*, *XPNPEP1*) were related genes added by GeneMANIA (Table 3).

### 3.4. Gene Ontology (GO) and Pathway Enrichment Analysis of Hub Genes

In order to gain insights into the biological roles of the hub genes, we performed GO enrichment analysis using Enrichr. We collected the GO annotations for 15 hub genes, including biological process (BP), molecular function (MF), and cellular process (CC). Furthermore, significant GO terms with a *p*-value ≤ 0.05 were selected.

**Figure 8:**
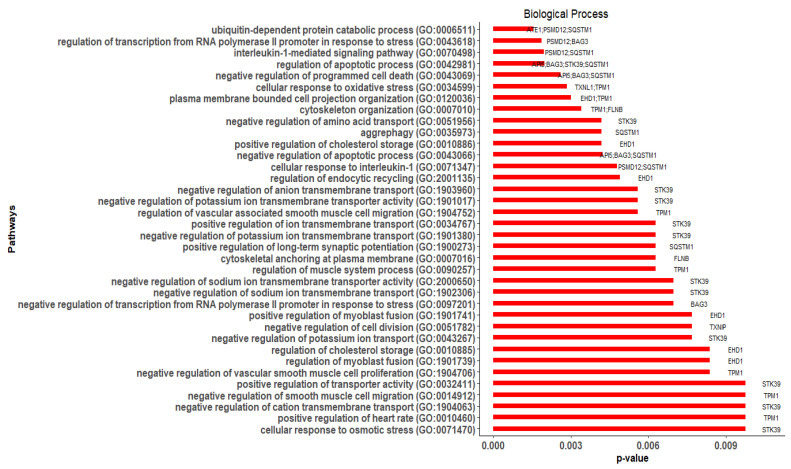
The significantly enriched hub genes in GO-BP (gene ontology-biological process) terms identified using Enrichr.

Of the 15 hub genes, 11 were significantly (*p*-value ≤ 0.05) enriched in 36 GO-BP terms (Figure 8). The enriched genes (and their respective counts) were *STK39* (12), *SQSTM1* (8), *TPM1* (8), *EHD1* (6), *BAG3* (5), *PSMD12* (4), *API5* (3), *FLNB* (2), *ATE1* (1), *TXNIP* (1), and *TXNL1* (1). Among them, *SQSTM1*, *EHD1*, *API5*, and *ATE1* were DEGs, while *STK39*, *TPM1*, *BAG3*, *PSMD12*, *FLNB*, *TXNIP*, and *TXNL1* were related genes added by GeneMANIA (Table 3). The BP terms that were enriched with more than one hub gene were regulation of apoptotic process [GO:0042981] (*API5*, *BAG3*, *STK39*, *SQSTM1*), negative regulation of programmed cell death [GO:0043069] (*API5*, *BAG3*, *SQSTM1*), negative regulation of apoptotic process [GO:0043066] (*API5*, *BAG3*, *SQSTM1*), interleukin-1-mediated signaling pathway [GO:0070498] (*PSMD12*, *SQSTM1*), regulation of transcription from RNA polymerase II promoter in response to stress [GO:0043618] (*PSMD12*, *BAG3*), cytoskeleton organization [GO:0007010] (*TPM1*, *FLNB*), and cellular response to oxidative stress [GO:0034599] (*TXNL1*, *TPM1*).

**Figure 9:**
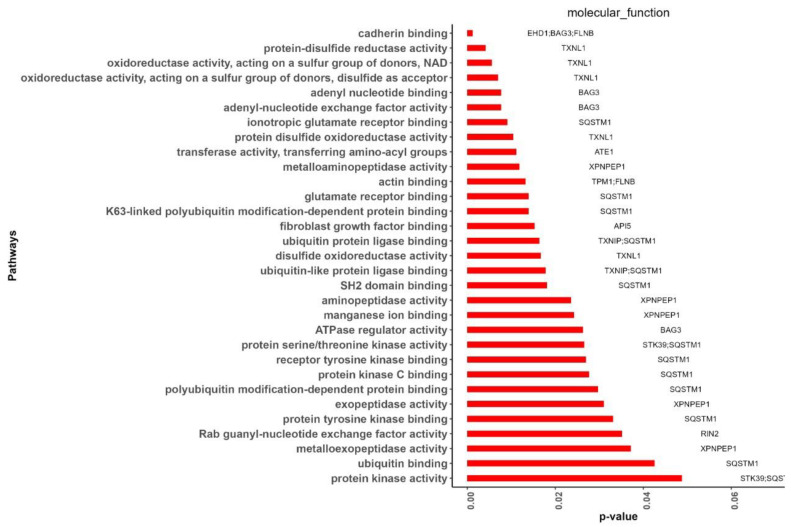
The significantly enriched hub genes in GO-MF (gene ontology-molecular function) terms identified using Enrichr.

Twelve of the fifteen hub genes were significantly (*p*-value ≤ 0.05) enriched in thirty-two GO-MF terms (Figure 9). The enriched genes were *SQSTM1* (13), *TXNL1* (5), *XPNPEP1* (5), *BAG3* (4), *FLNB* (2), *STK39* (2), *TXNIP* (2), *API5* (1), *ATE1* (1), *EHD1* (1), *RIN2* (1), and *TPM1* (1). Among them, *SQSTM1*, *API5*, *ATE1*, *EHD1*, and *RIN2* were DEGs, while *TXNL1*, *XPNPEP1*, *BAG3*, *FLNB*, *STK39*, *TXNIP*, and *TPM1* were related genes. The MF terms that were enriched with more than one hub gene were cadherin binding [GO:0045296] (*EHD1*, *BAG3*, *FLNB*), protein serine/threonine kinase activity [GO:0004674] (*STK39*, *SQSTM1*), protein kinase activity [GO:0004672] (*STK39*, *SQSTM1*), actin binding [GO:0003779] (*TPM1*, *FLNB*), ubiquitin protein ligase binding [GO:0031625] (*TXNIP*, *SQSTM1*), and ubiquitin-like protein ligase binding [GO:0044389] (*TXNIP*, *SQSTM1*).

**Figure 10:**
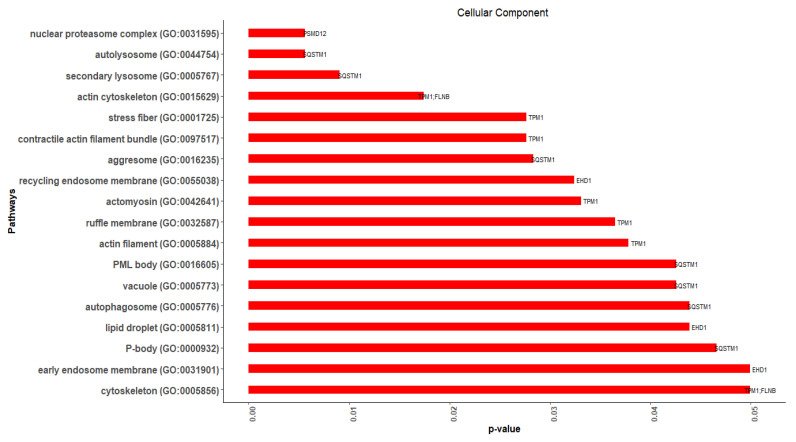
The significantly enriched hub genes in GO-CC (gene ontology-cellular component) terms identified using Enrichr.

In 18 GO-CC terms, 5 of the 15 hub genes were significantly (*p*-value ≤ 0.05) enriched (Figure 10). The enriched genes were *SQSTM1* (7), *TPM1* (7), *EHD1* (3), *FLNB* (2), and *PSMD12* (1). *SQSTM1* and *EHD1* were DEGs, while *TPM1*, *FLNB,* and *PSMD12* were related genes. Actin cytoskeleton [GO:0015629] (*TPM1*, *FLNB*), and cytoskeleton [GO:0005856] (*TPM1*, *FLNB*) were the CC terms that were enriched with more than one hub gene.

**Figure 11:**
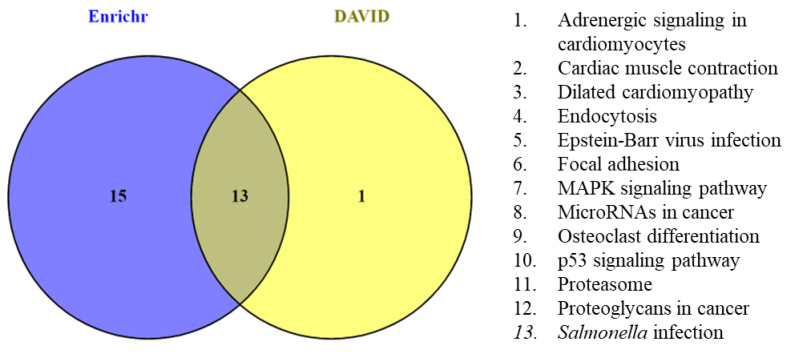
Venn diagram depicting the overlapped KEGG pathways identified using Enrichr and DAVID.

Furthermore, Enrichr-, and DAVID-based KEGG pathway enrichment analyses were performed to better understand the signaling pathway enrichment of hub genes. Enrichr revealed that 7 of the 15 hub genes were enriched in 28 KEGG pathways. The enriched genes were *PSMD12* (9), *SQSTM1* (9), *TPM1* (5), *FLNB* (4), *CCNG1* (2), *EHD1* (1), and *TXNIP* (1). This finding was further confirmed by DAVID, which showed that 6 genes were enriched in 14 KEGG pathways. The enriched genes were *TPM1* (5), *FLNB* (4), *CCNG1* (2), *PSMD12* (2), *EHD1* (1), and *SQSTM1* (1). A total of 6 hub genes *PSMD12*, *SQSTM1*, *TPM1*, *FLNB*, *CCNG1*, and *EHD1* were found to be overlapping in 13 pathways (Figure 11). Among the six genes, *SQSTM1*, *CCNG1*, and *EHD1* were DEGs, whereas *PSMD12*, *TPM1*, and *FLNB* were related genes. The overlapping pathways were adrenergic signaling in cardiomyocytes (*TPM1*), cardiac muscle contraction (*TPM1*), dilated cardiomyopathy (*TPM1*), endocytosis (*EHD1*), Epstein–Barr virus infection (*PSMD12*), focal adhesion (*FLNB*), MAPK signaling pathway (*FLNB*), microRNAs in cancer (*TPM1*), steoclast differentiation (*SQSTM1*), p53 signaling pathway (*CCNG1*), proteasome (*PSMD12*), proteoglycans in cancer (*FLNB*), and *Salmonella* infection (*FLNB*).

**Figure 12:**
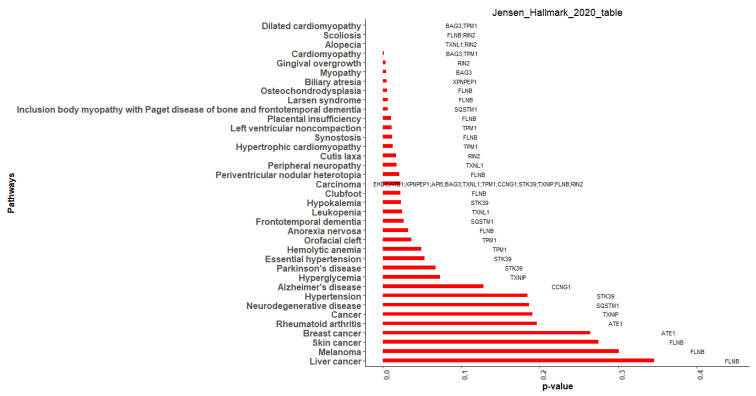
Enrichr-Jensen DISEASES analysis of hub genes.

Finally, Jensen DISEASES, an Enrichr feature that emphasizes links between genes and diseases, further supported the finding. Of the 15 hub genes, 13 were enriched in 25 Jensen DISEASES terms (Figure 12). The enriched genes were *FLNB* (9), *TPM1* (7), *RIN2* (5), *BAG3* (4), *TXNL1* (4), *SQSTM1* (2), *STK39* (2), *XPNPEP1* (2), *API5* (1), *ATE1*(1), *CCNG1* (1), *EHD1* (1), and *TXNIP* (1). Of the over-represented genes, *RIN2*, *SQSTM1*, *API5*, *ATE1*, *CCNG1*, and *EHD1* were DEGs, while *FLNB*, *TPM1*, *BAG3*, *TXNL1*, *STK39*, *XPNPEP1*, and *TXNIP* were related genes. The Enrichr-Jensen DISEASES terms that were enriched with more than one hub gene were carcinoma (*EHD1*, *ATE1*, *XPNPEP1*, *API5*, *BAG3*, *TXNL1*, *TPM1*, *CCNG1*, *STK39*, *TXNIP*, *FLNB*, *RIN2*), dilated cardiomyopathy (*BAG3*, *TPM1*), alopecia (*TXNL1*, *RIN2*), scoliosis (*FLNB*, *RIN2*), and cardiomyopathy (*BAG3*, *TPM1*).

## 4. Discussion

Rheumatoid arthritis (RA), osteoarthritis (OA), and periodontal disease (PD) are chronic inflammatory diseases that are globally prevalent and pose a public health concern. However, most of the research is concentrated on one disease at a time [54,55]. Recent advances in the study of RA and PD, OA and PD, RA and OA, as well as RA, OA, and PD separately, have been made by utilizing the data generated from high-throughput technologies (DNA microarrays and RNAseq), and Omics-based techniques [5,6,8,16,20,37,39,56,57,58]. However, their applicability is limited when it comes to identifying integrated signature molecules for RA, OA, and PD together. Macrophages play a significant role in disease progression in RA, OA, and PD [6,17,26,29,30,39,45,50,59,60]. A recent study revealed that PG can interfere with the host immune system by preventing intracellular apoptosis and inflammasome activation in macrophages [29]. This is done by modifying the lipopolysaccharide (LPS) composition in order to evade pattern recognition by phagocytes [19]. Apoptosis is involved in the removal of pathogens, diseased or damaged cells, as well as the development of inflammatory diseases via the PI3K/AKT/p53 signaling pathways. Bacterial resistance mechanisms cause anti-apoptosis proteins to invade the host defense system, implying that apoptosis inhibition is an important survival and pathogenicity mechanism [61]. PG or its constituents, such as fimbriae and LPS, act as danger signals, stimulating cell surface receptors such as Toll-like receptors (TLRs) [62] as well as Nod-like receptors (NLRs) [63]. Furthermore, lipids derived from PG can impair osteoblastic function, initiate inflammation, and ultimately alter osteoblast physiology [19,28,64].

OA alters the articular cartilage, synovium, and bone. Articular cartilage is a highly specialized connective tissue found in joints that are composed of chondrocytes. Chondrocytes maintain the structural and functional integrity of cartilage by synthesizing an extracellular matrix (ECM). This process is known as cartilage homeostasis. Hence, chondrocyte preservation is critical to joint health because articular cartilage lacks blood vessels and nerves and has a limited capacity for intrinsic repair. In response to OA stimuli, however, chondrocytes lose their ability to maintain cartilage integrity and their survival [65]. Inflamed OA synovium and damaged cartilage produce pro-inflammatory cytokines such as interleukin (IL)-1, tumor necrosis factor (TNF)-α, and IL-6 via paracrine or autocrine pathways, and together they activate NF-κB signaling pathways [65,66]. Due to restricted blood supply, limited ECM synthesis, and low cell density, cartilage regeneration is difficult and diminishes during prolonged inflammation [67]. Thus, overexpression of apoptotic inhibitors by PG or its component can be one of the possible mechanisms that explains the interplay of PG in OA. On the other hand, reduced expression of an apoptotic inhibitor results in an increase in apoptosis. Consistently circulating apoptotic debris can induce autoreactive cells and develop immunological complexes [68], which may play a significant part in RA. Since synovial fibroblasts of RA are the most severely impacted by apoptosis, it leads to gradual degradation of articular cartilage [18]. As apoptosis is increased during the early stages of infection and gradually decreases during the later stages of infection, we can assume that PG may be involved in RA during the early stages of infection and that the prolonged infection with PG may result in OA. Hence, analysis of PG-infected host macrophage and synovial macrophage gene expression profiles are required in order to identify the subcellular activities affected by this bacteria. 

In this study, we identified DEGs by using gene expression profiles from RA and PG-SM, as well as RNAseq data from OA. A total of 72 common DEGs (RA and PG; Standard Error = 0.012) were found across the data sets, with 47 of them being highly correlated based on their r-values. A GeneMANIA interaction network for the highly correlated DEGs comprised 53 and 271 nodes and edges, respectively (Figure 6). Among the 53 nodes, 33 were DEGs, while the remaining 20 were related genes added by GeneMANIA (Table 3). Network centrality analysis identified 15 hub genes, of which 6 genes (*API5*, *ATE1*, *CCNG1*, *EHD1*, *RIN2*, and *SQSTM1*) were DEGs while the remaining 9 (*ATP6V1E1*, *BAG3*, *FLNB*, *PSMD12*, *STK39*, *TPM1*, *TXNIP*, *TXNL1*, and *XPNPEP1*) were related genes.

The differential gene expression analysis showed that *API5*, *ATE1*, *CCNG1*, *EHD1*, *IER3*, and *RGS16* were significantly dysregulated in all three macrophage conditions. Two DEGs, *RGS16* (RA, logFC = 1.141; OA, logFC = 2.358; PG, logFC = 1.065) and *IER3* (RA, logFC = 1.583; OA, logFC = 1.611; PG, logFC = 3.691), were considerably elevated in all macrophage conditions as compared to other DEGs which were either up/down-regulated. The GeneMANIA interaction network showed that *RGS16* is linked to hub genes *CCNG1* and *TXNIP*, whereas *IER3* is linked to hub genes *ATE1*, *EHD1*, *SQSTM1*, and *BAG1*. Both *RGS16* and *IER3* are associated with the IL-18 signaling pathway, which is known to influence the activity of osteoclasts and osteoblasts/chondrocytes, and may operate as a physiological regulator of bone development [69]. Therefore dysregulation of the IL-18 signaling pathway plays a major role in RA [69] and OA [70]. The IL-18 signaling pathway also plays an important role in pro-inflammatory cytokine production in PD, which is the main cause of alveolar bone loss in the presence of PG [71]. Therefore, even though *IER3* and *RGS16* are non-hub genes, it is crucial to understand their role in three macrophage conditions.

Interestingly, hub genes *SQSTM1*, *FLNB*, *TXNL1*, and *ATP6V1E1* had previously been related to one or more of the three diseases. *SQSTM1* (sequestosome1) is already known to play a critical role in the evolution of OA via microRNAs (miRNAs). It also inhibits cytokine expression in activated macrophages [72,73]. *FLNB* (filamin B) plays a role in the spondylocarpotarsal synostosis (SCT) syndrome, Larsen syndrome, atelosteogenesis types I and III (AOI and AOIII), and Piepkorn osteochondrodysplasia (POCD), which are also associated with the bone disorders [74]. *FLNB* is largely expressed in macrophages, and governs skeletal development by promoting the migration, proliferation, and secretion of pro-inflammatory cytokines as well as the synthesis of metallopeptidase-9 (*MMP-9*) and extracellular signal-regulated kinase (*ERK*) activity. Dysregulation of *FLNB* leads to various bone diseases. Recently, *FLNB* has been linked to periodontal ligament and bone marrow development [74,75,76,77,78,79]. *TXNL1* (thioredoxin-like 1) is a redox-scavenger gene involved in the management of oxidative stress, which is a critical factor in the development of RA, OA, and PG [79]. *ATP6V1E1* (ATPase H+ transporting V1 subunit E1) is associated with osteoclastic activity, which destroys joints and affects inflammatory responses such as phagocytosis, cytokine secretion, and neutrophil granule exocytosis [80]. This shows that the related genes added by GeneMANIA are not random, but play a crucial role in the aetiology of RA, OA, and PD.

*API5* (apoptosis inhibitor 5) is down-regulated in OA (logFC = −1.612) and PG (logFC = −0.838), but up-regulated in RA (logFC = 0.949). *API5* binds to TLR4 and activates TLR signaling pathways, hence activating NF-kB [81]. NF-kB dysregulation has been linked to inflammatory and autoimmune diseases [82], as well as the disruption of cartilage homeostasis that leads to OA [65] and RA [83]. *API5* under-expression is linked to the pathogenesis of systemic lupus erythematosus (SLE) via apoptosis [84], whereas overexpression is linked to the progression of various cancers [65].

*ATE1* (Arginyltransferase 1) is down-regulated in RA (logFC = −0.683), OA (logFC = −0.909), and PG (logFC = −1.018). *ATE1* catalyzes the post-translational conjugation of arginine to the N-terminal aspartate or glutamate of a protein required for ubiquitin-mediated degradation. Furthermore, down-regulation of *ATE1* allows bacteria to develop in the presence of stress stimuli, such as oxidative stress or high nitric oxide concentrations, which can induce cell growth inhibition or cell death, as well as chronic inflammation and resistance. Numerous studies have shown that sufficient arginylation is required for anti-apoptotic action in the presence of an apoptotic inhibitor as well as a protein that destroys pro-inhibitors or similar proteins, such as G-coupled protein regulators [85,86] 

*EHD1* (EH Domain Containing 1) is a member of a conserved gene family that produces EH domain-containing proteins, and is overexpressed in RA (logFC = 2.131) and PG (logFC = 2.084), but underexpressed in OA (logFC = −2.591). It controls phosphophagocytosis, cytokine secretion, cell proliferation, and motility, as well as macrophage receptor activation and signaling [87]. *EHD1* also regulates protein–protein interactions, intracellular sorting, and IGF1 receptor endocytosis. *EHD1* mediates *IGF1* receptor endocytosis through an adaptor protein complex. *IGF1* is important in cartilage metabolism; it promotes chondrocyte proliferation, matrix protein production, terminal differentiation, and mineralization, as well as raising cellular calcium levels [88]. *EHD1* dysregulation impairs IGF1-mediated signaling, which results in RA and OA [79]. PG is also known to increase IGF1 expression [89], and one possible explanation could be *EHD1* dysregulation, which leads to RA and OA.

*CCNG1* (Cyclin G1) is a topologically significant gene with the highest bottleneck value (BN: 7) and is underexpressed in all three macrophage conditions (RA, logFC = −0.712; OA, logFC = −1.613; PG, logFC = −0.784). *CCNG1* regulates cell proliferation, apoptosis, migration, invasion, and cell cycle arrest. Inhibiting *CCNG1* activates p53 and causes apoptosis [90]. PG can regulate cell cycle disruption and apoptosis through the involvement of p53 [91]. p53 regulates *CCNG1* expression, and its dysregulation is linked to osteosarcoma (bone cancer) [92]. Furthermore, *CCNG1* regulates mesenchymal stem cell (MSC) differentiation, and the absence or loss of p53 causes abnormal osteogenic differentiation of MSCs, which results in imbalanced bone remodeling [93]. As a bottleneck gene, *CCNG1* may be an important link in explaining the role of PG in RA and OA.

For *RIN2* (Ras and Rab interactor 2), Enrichr-Jensen DISEASES analysis associated *RIN2* with gingival overgrowth, and literature studies associate it with endocytosis and bone formation [94]. *RIN2*, a widely expressed protein, interacts with Rab5 to regulate endocytic trafficking, as well as to target and fuse endocytic vesicles to early endosomes [95]. The activation of Rab5 via TNF-α increases the ability of PG to invade human gingival epithelial cells [96].

We also examined the importance of the related gene *STK39* (serine/threonine kinase 39), which appears to be significant based on topological parameters (DC:19, BC:139.88; BN:2; CC:34.33). It is involved in the stress response pathway, actively regulating the p38 MAP kinase pathway and TCR signaling in naive CD4+ T cells. It is well-known for its role in inflammatory diseases, macrophage activation, and bacterial growth [97,98]. According to research, *STK39* dysregulation affects *MMP2* (matrix metallopeptidase 2) and *MMP9* (matrix metallopeptidase 9), thus actively participating in osteosarcoma [99]. As a result, it may play an important role in other bone-related diseases such as RA, OA, and PD.

The major limitation of our study is that the data we collected are from different years and each data set has a methodological constraint of being generated using different protocols. Despite these differences, the variances are reasonably low. Our results should be interpreted in terms of variance among the data.

## 5. Conclusion

The results of the present study proposed 8-gene signatures including *API5*, *ATE1*, *CCNG1*, *EHD1*, *IER3*, *RGS16*, *RIN2*, and *STK39* by employing gene expressions and network-based studies. These gene signatures could aid in the early diagnoses of RA, OA, and PD; however, experimental studies are needed to confirm these findings.

## Figures and Tables

**Table 1:** Details of the gene expression data retrieved from the GEO (Gene Expression Omnibus) database for rheumatoid arthritis (RA), osteoarthritis (OA), and *Porphyromonas gingivalis* (PG).

Disease	Series ID	Description	No. of Samples	Platform	Year	References
RA	GSE97779	Expression data from RA synovial macrophages	14	GPL570 (Affymetrix Human Genome U133 Plus 2.0 Array)	2017	[39]
OA	GSE123492	RNA sequencing of highly pure synovial tissue macrophages reveals two distinct OA subgroups that indicate different disease mechanisms.	9	GPL18573 (Illumina NextSeq 500)	2019	[6]
PG	GSE24897	Expression data from human macrophages treated with PG and its components.	6	GPL570 (Affymetrix Human Genome U133 Plus 2.0 Array)	2010	[20]

**Table 2:** List of highly correlated (r score > 0.7) differentially expressed genes (DEGs) in rheumatoid arthritis (RA), osteoarthritis (OA), and *Porphyromonas gingivalis* (PG) macrophages, along with their log2-fold change (logFC) ≥ ±1 and *p*-value ≤ 0.05.

S.No.	Genes		RA			OA			PG	
		r-Value	logFC	*p*-Value	r-Value	logFC	*p*-Value	r-Value	logFC	*p*-Value
1	*ABHD5*	0.88	−0.566	0.002	0.9101	−3.106	0.02	0.9866	−0.707	0.001
2	*AKAP13*	0.839	1.093	0	0.9723	2.119	0.018	0.9253	0.925	0
3	*API5*	0.7908	0.949	0	0.9587	−1.612	0.044	0.9681	−0.838	0.004
4	*ATE1*	0.8574	−0.683	0	0.9346	−0.909	0.011	0.9726	−1.018	0.002
5	*ATP1B1*	0.7904	−1.457	0.003	0.8849	3.119	0.027	0.9819	0.777	0.001
6	*ATPAF1*	0.773	−0.656	0.006	0.9537	−1.314	0.004	0.9896	−0.898	0.002
7	*BLOC1S2*	0.8767	−1.199	0	0.9725	−0.662	0.018	0.981	−0.648	0
8	*CAB39L*	0.9258	−1.924	0	0.9697	4.238	0.003	0.9658	−1.231	0
9	*CCDC66*	0.8811	−0.65	0.002	0.8958	−3.632	0.004	0.9899	−0.96	0
10	*CCNG1*	0.8742	−0.712	0	0.923	−1.613	0.009	0.9745	−0.784	0.001
11	*CISD1*	0.847	−1.034	0.004	0.9385	−2.788	0.031	0.9263	−0.809	0.002
12	*CLIP4*	0.8257	−0.648	0.003	0.9052	−3.548	0.011	0.9836	−1.127	0
13	*DAGLB*	0.7146	1.117	0.002	0.9608	1.844	0.007	0.9891	−0.768	0
14	*EHD1*	0.8456	2.131	0	0.8832	−2.591	0.041	0.9949	2.084	0
15	*ERCC1*	0.8019	0.92	0.001	0.9718	−2.896	0.016	0.9864	−0.647	0
16	*ETFA*	0.9509	−0.628	0	0.925	1.39	0.015	0.9891	−0.528	0.003
17	*FH*	0.8747	−0.647	0	0.9047	−3.761	0.007	0.9754	−1.096	0.002
18	*FMNL1*	0.9714	1.529	0	0.9816	−2.894	0.027	0.9806	1.036	0
19	*GGCT*	0.8915	−0.863	0	0.9109	2.605	0.012	0.9855	−0.758	0
20	*HNMT*	0.8874	−1.133	0	0.8968	−1.428	0.007	0.9967	−0.822	0.001
21	*IER3*	0.9039	1.583	0	0.8884	1.611	0.036	0.99	3.691	0
22	*KBTBD7*	0.9135	−3.025	0	0.8291	1.669	0.041	0.9924	−0.899	0.002
23	*KDM7A*	0.8482	1.082	0	0.948	2.185	0.047	0.9788	0.819	0.003
24	*KLHL28*	0.8184	−0.857	0	0.8628	3.24	0.035	0.9868	1.469	0
25	*MBTPS2*	0.8484	−0.604	0	0.8632	−1.722	0	0.9795	−0.779	0.003
26	*MCEE*	0.8159	−0.739	0	0.7189	0.793	0.021	0.991	−1.114	0
27	*METTL25*	0.7534	−0.631	0.001	0.9246	0.725	0.038	0.9649	−0.708	0.003
28	*MTIF3*	0.8244	−0.783	0.001	0.9307	−0.618	0.046	0.9964	−1.215	0
29	*NLRP3*	0.8997	4.303	0	0.8854	−1.11	0.017	0.9812	2.918	0
30	*PMEPA1*	0.7735	1.412	0.001	0.8759	−2.231	0.042	0.9633	0.656	0.001
31	*PNRC1*	0.8301	0.597	0.007	0.7468	1.758	0.041	0.9967	1.881	0
32	*PPP1R18*	0.7838	0.598	0.001	0.9788	1.476	0.047	0.9778	1.106	0
33	*PSTPIP1*	0.8447	1.879	0	0.9272	1.495	0.045	0.9646	−0.819	0
34	*RCBTB2*	0.8567	−0.788	0	0.8104	1.973	0.048	0.9772	−1.386	0.001
35	*RGS16*	0.7793	1.141	0.006	0.8896	2.358	0.022	0.9743	1.065	0
36	*RHNO1*	0.8083	−0.592	0.001	0.8469	2.126	0.031	0.9891	−1.008	0.001
37	*RIN2*	0.8317	3.051	0	0.7911	−1.47	0.042	0.9943	−1.559	0.001
38	*RPAP3*	0.8334	−0.54	0.003	0.719	−0.786	0.012	0.99	−1.598	0
39	*RPGR*	0.8102	0.651	0.002	0.816	2.703	0.044	0.968	0.724	0
40	*RPUSD3*	0.8895	−1.102	0	0.8743	−0.585	0.046	0.9632	−1.082	0
41	*SAMHD1*	0.8755	0.653	0	0.9581	−3.903	0.017	0.9696	−1.287	0.004
42	*SAR1B*	0.9441	−0.739	0	0.9027	0.843	0	0.993	−0.786	0
43	*SLC20A1*	0.9401	0.572	0	0.957	−1.416	0.013	0.997	−0.775	0.001
44	*SQSTM1*	0.9476	−1.311	0	0.9383	−2.452	0.03	0.9756	0.907	0.003
45	*SYNRG*	0.8561	−0.599	0.005	0.8443	−2.746	0.007	0.9649	−0.765	0
46	*TAPBP*	0.8511	0.913	0	0.9346	2.118	0.03	0.976	0.909	0.001
47	*TBC1D12*	0.8615	−0.738	0.001	0.8791	0.655	0.004	0.9303	0.546	0.001

**Table 3:** List of top 15 hub genes in the interaction network identified using the Cytoscape plugin cytoHubba. query = DEG, result = related gene, DC = degree centrality, BC = betweenness centrality, BN = bottleneck, and CC = closeness centrality.

S.No.	Gene Name	Node Type	DC	BC	BN	CC
1	*API5*	query	21	226.67	7	34.00
2	*ATE1*	query	14	71.11	1	32.00
3	*ATP6V1E1*	result	13	114.54	1	31.17
4	*BAG3*	result	16	94.49	2	32.50
5	*CCNG1*	query	14	79.93	7	31.83
6	*EHD1*	query	17	166.16	4	33.33
7	*FLNB*	result	20	122.38	2	34.17
8	*PSMD12*	result	15	61.81	2	32.17
9	*RIN2*	query	17	138.44	1	33.17
10	*SQSTM1*	query	16	72.99	4	31.33
11	*STK39*	result	19	139.88	2	34.33
12	*TPM1*	result	18	99.21	1	33.17
13	*TXNIP*	result	15	95.66	4	32.67
14	*TXNL1*	result	17	155.47	2	33.50
15	*XPNPEP1*	result	18	108.06	3	33.33

## Data Availability

The data sets used to support the findings of this study are included within the article and Supplementary Information Files.

## Supplementary Materials

The following supporting information can be downloaded at: https://www.mdpi.com/article/10.3390/cimb44080241/s1, Table S1: List of 72 common differentially expressed genes (DEGs) among rheumatoid arthritis (RA), osteoarthritis (OA), and *Porphyromonas gingivalis* (PG), Table S2: GeneMANIA interaction network between the 47 highly correlated common differentially expressed genes (DEGs).

## References

[1] Deodhar A.A., Woolf A.D. (1996). Bone mass measurement and bone metabolism in rheumatoid arthritis: A review. Br. J. Rheumatol..

[2] Firestein G.S. (2003). Evolving concepts of rheumatoid arthritis. Nature.

[3] Chen B., Zhao Y., Li S., Yang L., Wang H., Wang T., Bin S., Gai Z., Heng X., Zhang C. (2018). Variations in oral microbiome profiles in rheumatoid arthritis and osteoarthritis with potential biomarkers for arthritis screening. Sci. Rep..

[4] Favazzo L.J., Hendesi H., Villani D.A., Soniwala S., Dar Q.-A., Schott E.M., Gill S.R., Zuscik M.J. (2020). The gut microbiome-joint connection: Implications in osteoarthritis. Curr. Opin. Rheumatol..

[5] Kim H.-S., Park H.-M., Kim H., Lee H.S., Son D.-H., Lee Y.-J. (2022). Association Between the Severity of Periodontitis and Osteoarthritis in Middle-Aged and Older Patients With Type 2 Diabetes Mellitus: A Nationwide Population-Based Study. Arthritis Care Res..

[6] Wood M.J., Leckenby A., Reynolds G., Spiering R., Pratt A.G., Rankin K.S., Isaacs J.D., Haniffa M.A., Milling S., Hilkens C.M. (2019). Macrophage proliferation distinguishes 2 subgroups of knee osteoarthritis patients. JCI Insight.

[7] Huang Z.Y., Stabler T., Pei F.X., Kraus V.B. (2016). Both systemic and local lipopolysaccharide (LPS) burden are associated with knee OA severity and inflammation. Osteoarthr. Cartil..

[8] Zuo B., Zhu J., Xiao F., Wang C., Shen Y., Chen X. (2020). Identification of novel biomarkers and candidate small molecule drugs in rheumatoid arthritis and osteoarthritis based on bioinformatics analysis of high-throughput data. Biosci. Rep..

[9] Suzuki A., Horie T., Numabe Y. (2019). Investigation of molecular biomarker candidates for diagnosis and prognosis of chronic periodontitis by bioinformatics analysis of pooled microarray gene expression datasets in Gene Expression Omnibus (GEO). BMC Oral Health.

[10] Sao P., Vats S., Singh S. (2022). *Porphyromonas gingivalis* resistance and virulence: An integrated functional network analysis. Gene.

[11] Sao P., Chand Y., Kumar A., Singh S. (2021). Potential Drug Target Identification in *Porphyromonas gingivalis* using In-silico Subtractive Metabolic Pathway Analysis. Bangladesh J. Med. Sci..

[12] Beck J.D., Papapanou P.N., Philips K.H., Offenbacher S. (2019). Periodontal Medicine: 100 Years of Progress. J. Dent. Res..

[13] Friedewald V.E., Kornman K.S., Beck J.D., Genco R., Goldfine A., Libby P., Offenbacher S., Ridker P.M., Dyke T.E.V., Roberts W.C. (2009). The American Journal of Cardiology and Journal of Periodontology Editors’ Consensus: Periodontitis and Atherosclerotic Cardiovascular Disease. Am. J. Cardiol..

[14] Lamster I.B., Lalla E., Borgnakke W.S., Taylor G.W. (2008). The Relationship Between Oral Health and Diabetes Mellitus. J. Am. Dent. Assoc..

[15] Lundmark A., Davanian H., Båge T., Johannsen G., Koro C., Lundeberg J., Yucel-Lindberg T. (2015). Transcriptome analysis reveals mucin 4 to be highly associated with periodontitis and identifies pleckstrin as a link to systemic diseases. Sci. Rep..

[16] Perricone C., Ceccarelli F., Matteo S., Di Carlo G., Bogdanos D.P., Lucchetti R., Pilloni A., Valesini G., Polimeni A., Conti F. (2019). *Porphyromonas gingivalis* and rheumatoid arthritis. Curr. Opin. Rheumatol..

[17] Totaro M.C., Cattani P., Ria F., Tolusso B., Gremese E., Fedele A.L., D’Onghia S., Marchetti S., Sante G.D., Canestri S. (2013). *Porphyromonas gingivalis* and the pathogenesis of rheumatoid arthritis: Analysis of various compartments including the synovial tissue. Arthritis Res. Ther..

[18] Röhner E., Detert J., Kolar P., Hocke A., N’Guessan P., Matziolis G., Kanitz V., Bernimoulin J.P., Kielbassa A., Burmester G.R. (2010). Induced apoptosis of chondrocytes by *Porphyromonas gingivalis* as a possible pathway for cartilage loss in rheumatoid arthritis. Calcif. Tissue Int..

[19] Blasi I., Korostoff J., Dhingra A., Reyes-Reveles J., Shenker B.J., Shahabuddin N., Alexander D., Lally E.T., Bragin A., Boesze-Battaglia K. (2016). Variants of *Porphyromonas gingivalis* lipopolysaccharide alter lipidation of autophagic protein, microtubule-associated protein 1 light chain 3, LC3. Mol. Oral Microbiol..

[20] Yu W.-H., Hu H., Zhou Q., Xia Y., Amar S. (2010). Bioinformatics Analysis of Macrophages Exposed to *Porphyromonas gingivalis*: Implications in Acute vs. Chronic Infections. PLoS ONE.

[21] Weinberg J.B., Wortham T.S., Misukonis M.A., Patton K.L., Chitneni S.R. (1993). Synovial mononuclear phagocytes in rheumatoid arthritis and osteoarthritis: Quantitative and functional aspects. Immunol. Investig..

[22] Kennedy A., Fearon U., Veale D.J., Godson C. (2011). Macrophages in Synovial Inflammation. Front. Immunol..

[23] Raggi F., Pelassa S., Pierobon D., Penco F., Gattorno M., Novelli F., Eva A., Varesio L., Giovarelli M., Bosco M.C. (2017). Regulation of Human Macrophage M1–M2 Polarization Balance by Hypoxia and the Triggering Receptor Expressed on Myeloid Cells-1. Front. Immunol..

[24] Werheim E.R., Senior K.G., Shaffer C.A., Cuadra G.A. (2020). Oral Pathogen *Porphyromonas gingivalis* Can Escape Phagocytosis of Mammalian Macrophages. Microorganisms.

[25] Papadopoulos G., Shaik-Dasthagirisaheb Y.B., Huang N., Viglianti G.A., Henderson A.J., Kantarci A., Gibson F.C. (2017). Immunologic Environment Influences Macrophage Response to *Porphyromonas gingivalis*. Mol. Oral Microbiol..

[26] Kurowska-Stolarska M., Alivernini S. (2017). Synovial tissue macrophages: Friend or foe?. RMD Open.

[27] Montgomery A., Fahy N., Hamilton S., Eckman B., Almeida L.D., Ishihara S., Mayr M.G., Chen S.Y., Gadhvi G., Cuda C. (2020). Macrophages drive the inflammatory phase in experimental osteoarthritis. bioRxiv.

[28] Zhao Y., Chen B., Li S., Yang L., Zhu D., Wang Y., Wang H., Wang T., Shi B., Gai Z. (2018). Detection and characterization of bacterial nucleic acids in culture-negative synovial tissue and fluid samples from rheumatoid arthritis or osteoarthritis patients. Sci. Rep..

[29] Kassem A., Henning P., Lundberg P., Souza P.P.C., Lindholm C., Lerner U.H. (2015). *Porphyromonas gingivalis* Stimulates Bone Resorption by Enhancing RANKL (Receptor Activator of NF-κB Ligand) through Activation of Toll-like Receptor 2 in Osteoblasts. J. Biol. Chem..

[30] Marchesan J.T., Gerow E.A., Schaff R., Taut A.D., Shin S.-Y., Sugai J., Brand D., Burberry A., Jorns J., Lundy S.K. (2013). *Porphyromonas gingivalis* oral infection exacerbates the development and severity of collagen-induced arthritis. Arthritis Res. Ther..

[31] Snijesh V.P., Matchado M.S., Singh S. (2018). Classifying Rheumatoid Arthritis gene network signatures for identifying key regulatory molecules and their altered pathways by adopting network biology approach. Gene Rep..

[32] Baliban R.C., Sakellari D., Li Z., DiMaggio P.A., Garcia B.A., Floudas C.A. (2012). Novel protein identification methods for biomarker discovery via a proteomic analysis of periodontally healthy and diseased gingival crevicular fluid samples. J. Clin. Periodontol..

[33] Singh S., Vennila J.J., Snijesh V.P., George G., Sunny C. (2016). Implying Analytic Measures for Unravelling Rheumatoid Arthritis Significant Proteins Through Drug–Target Interaction. Interdiscip. Sci. Comput. Life Sci..

[34] Barrett T., Edgar R. (2006). Mining microarray data at NCBI’s Gene Expression Omnibus (GEO)*. Methods Mol. Biol. Clifton NJ.

[35] Chand Y., Sao P., Singh S., Chandra N., Das S., Singh S. (2020). Prioritizing potential diagnostic biomarkers of Alzheimer’s disease by investigating gene expression data: A network-based approach. Alzheimers Dement..

[36] Sao P., Kishore I., Singh S., Panneerselvam K., Kumar A., Chaudhuri T. (2013). Putative Target Identification for Gout; A Network Biology Approach. J. Bionanosci..

[37] Singh S. (2015). Network Biology Approach for Identifying Significant Drug Targets and Pathways for Rheumatoid Arthritis.

[38] Taye B., Vaz C., Tanavde V., Kuznetsov V.A., Eisenhaber F., Sugrue R.J., Maurer-Stroh S. (2017). Benchmarking selected computational gene network growing tools in context of virus-host interactions. Sci. Rep..

[39] Kang K., Park S.H., Chen J., Qiao Y., Giannopoulou E., Berg K., Hanidu A., Li J., Nabozny G., Kang K. (2017). Interferon-γ Represses M2 Gene Expression in Human Macrophages by Disassembling Enhancers Bound by the Transcription Factor MAF. Immunity.

[40] Davis S., Meltzer P.S. (2007). GEOquery: A bridge between the Gene Expression Omnibus (GEO) and BioConductor. Bioinforma. Oxf. Engl..

[41] Ritchie M.E., Phipson B., Wu D., Hu Y., Law C.W., Shi W., Smyth G.K. (2015). Limma powers differential expression analyses for RNA-sequencing and microarray studies. Nucleic Acids Res..

[42] Smyth G.K. (2004). Linear Models and Empirical Bayes Methods for Assessing Differential Expression in Microarray Experiments. Stat. Appl. Genet. Mol. Biol..

[43] George G., Singh S., Lokappa S.B., Varkey J. (2019). Gene co-expression network analysis for identifying genetic markers in Parkinson’s disease-a three-way comparative approach. Genomics.

[44] Shannon P., Markiel A., Ozier O., Baliga N.S., Wang J.T., Ramage D., Amin N., Schwikowski B., Ideker T. (2003). Cytoscape: A software environment for integrated models of biomolecular interaction networks. Genome Res..

[45] Montojo J., Zuberi K., Rodriguez H., Bader G.D., Morris Q. (2014). GeneMANIA: Fast gene network construction and function prediction for Cytoscape. F1000Research.

[46] Blessia T.F., Singh S., Vennila J.J. (2017). Unwinding the Novel Genes Involved in the Differentiation of Embryonic Stem Cells into Insulin-Producing Cells: A Network-Based Approach. Interdiscip. Sci. Comput. Life Sci..

[47] Scardoni G., Lau C., Zhang Y. (2012). Centralities Based Analysis of Complex Networks. New Frontiers in Graph Theory.

[48] He X., Zhang J. (2006). Why Do Hubs Tend to Be Essential in Protein Networks?. PLoS Genet..

[49] Chin C.-H., Chen S.-H., Wu H.-H., Ho C.-W., Ko M.-T., Lin C.-Y. (2014). cytoHubba: Identifying hub objects and sub-networks from complex interactome. BMC Syst. Biol..

[50] Yu H., Kim P.M., Sprecher E., Trifonov V., Gerstein M. (2007). The Importance of Bottlenecks in Protein Networks: Correlation with Gene Essentiality and Expression Dynamics. PLoS Comput. Biol..

[51] Chen E.Y., Tan C.M., Kou Y., Duan Q., Wang Z., Meirelles G.V., Clark N.R., Ma’ayan A. (2013). Enrichr: Interactive and collaborative HTML5 gene list enrichment analysis tool. BMC Bioinform..

[52] Huang D.W., Sherman B.T., Tan Q., Kir J., Liu D., Bryant D., Guo Y., Stephens R., Baseler M.W., Lane H.C. (2007). DAVID Bioinformatics Resources: Expanded annotation database and novel algorithms to better extract biology from large gene lists. Nucleic Acids Res..

[53] Pletscher-Frankild S., Pallejà A., Tsafou K., Binder J.X., Jensen L.J. (2015). DISEASES: Text mining and data integration of disease–gene associations. Methods.

[54] Lorenzo D., GianVincenzo Z., Carlo Luca R., Karan G., Villafañe J.H., Roberto M., Javad P. (2019). Oral–Gut Microbiota and Arthritis: Is There an Evidence-Based Axis?. J. Clin. Med..

[55] Tong Y., Zheng L., Qing P., Zhao H., Li Y., Su L., Zhang Q., Zhao Y., Luo Y., Liu Y. (2020). Oral Microbiota Perturbations Are Linked to High Risk for Rheumatoid Arthritis. Front. Cell. Infect. Microbiol..

[56] Aihaiti Y., Tuerhong X., Ye J.-T., Ren X.-Y., Xu P. (2020). Identification of pivotal genes and pathways in the synovial tissue of patients with rheumatoid arthritis and osteoarthritis through integrated bioinformatic analysis. Mol. Med. Rep..

[57] Puentes-Osorio Y., Amariles P., Calleja M.Á., Merino V., Díaz-Coronado J.C., Taborda D. (2021). Potential clinical biomarkers in rheumatoid arthritis with an omic approach. Autoimmun. Highlights.

[58] Trindade F., Oppenheim F.G., Helmerhorst E.J., Amado F., Gomes P.S., Vitorino R. (2014). Uncovering the molecular networks in periodontitis. Proteom.-Clin. Appl..

[59] Hamerman J.A., Pottle J., Ni M., He Y., Zhang Z.-Y., Buckner J.H. (2016). Negative regulation of TLR signaling in myeloid cells—Implications for autoimmune diseases. Immunol. Rev..

[60] Liu X.-R., Xu Q., Xiao J., Deng Y.-M., Tang Z.-H., Tang Y.-L., Liu L.-S. (2020). Role of oral microbiota in atherosclerosis. Clin. Chim. Acta Int. J. Clin. Chem..

[61] de Andrade K.Q., Almeida-da-Silva C.L.C., Coutinho-Silva R. (2019). Immunological Pathways Triggered by *Porphyromonas gingivalis* and Fusobacterium nucleatum: Therapeutic Possibilities?. Mediat. Inflamm..

[62] How K.Y., Song K.P., Chan K.G. (2016). *Porphyromonas gingivalis*: An Overview of Periodontopathic Pathogen below the Gum Line. Front. Microbiol..

[63] Johnson L., Atanasova K.R., Bui P.Q., Lee J., Hung S.-C., Yilmaz Ö., Ojcius D.M. (2015). *Porphyromonas gingivalis* attenuates ATP-mediated inflammasome activation and HMGB1 release through expression of a nucleoside-diphosphate kinase. Microbes Infect..

[64] Wang M., Xie J., Wang C., Zhong D., Xie L., Fang H. (2020). Immunomodulatory Properties of Stem Cells in Periodontitis: Current Status and Future Prospective. Stem Cells Int..

[65] Choi M.-C., Jo J., Park J., Kang H.K., Park Y. (2019). NF-κB Signaling Pathways in Osteoarthritic Cartilage Destruction. Cells.

[66] Mathiessen A., Conaghan P.G. (2017). Synovitis in osteoarthritis: Current understanding with therapeutic implications. Arthritis Res. Ther..

[67] Yin W., Xu H., Sheng J., Xu Z., Xie X., Zhang C. (2017). Comparative evaluation of the effects of platelet-rich plasma formulations on extracellular matrix formation and the NF-κB signaling pathway in human articular chondrocytes. Mol. Med. Rep..

[68] Mok C.C., Lau C.S. (2003). Pathogenesis of systemic lupus erythematosus. J. Clin. Pathol..

[69] Cornish J., Gillespie M.T., Callon K.E., Horwood N.J., Moseley J.M., Reid I.R. (2003). Interleukin-18 Is a Novel Mitogen of Osteogenic and Chondrogenic Cells. Endocrinology.

[70] Fu Z., Liu P., Yang D., Wang F., Yuan L., Lin Z., Jiang J. (2012). Interleukin-18-induced inflammatory responses in synoviocytes and chondrocytes from osteoarthritic patients. Int. J. Mol. Med..

[71] Zhang Y., Kuang W., Li D., Li Y., Feng Y., Lyu X., Huang G.-B., Lian J.-Q., Yang X.-F., Hu C. (2021). Natural Killer-Like B Cells Secreting Interleukin-18 Induces a Proinflammatory Response in Periodontitis. Front. Immunol..

[72] Li H., Miao D., Zhu Q., Huang J., Lu G., Xu W. (2018). MicroRNA-17-5p contributes to osteoarthritis progression by binding p62/SQSTM1. Exp. Ther. Med..

[73] Mylka V., Deckers J., Ratman D., De Cauwer L., Thommis J., De Rycke R., Impens F., Libert C., Tavernier J., Vanden Berghe W. (2018). The autophagy receptor SQSTM1/p62 mediates anti-inflammatory actions of the selective NR3C1/glucocorticoid receptor modulator compound A (CpdA) in macrophages. Autophagy.

[74] Robertson S., Adam M.P., Ardinger H.H., Pagon R.A., Wallace S.E., Bean L.J., Mirzaa G., Amemiya A. (1993). FLNB Disorders. GeneReviews^®^.

[75] Dong R., Du J., Wang L., Wang J., Ding G., Wang S., Fan Z. (2014). Comparison of Long Noncoding RNA and mRNA Expression Profiles in Mesenchymal Stem Cells Derived from Human Periodontal Ligament and Bone Marrow. BioMed Res. Int..

[76] Lamsoul I., Métais A., Gouot E., Heuzé M.L., Lennon-Duménil A.-M., Moog-Lutz C., Lutz P.G. (2013). ASB2α regulates migration of immature dendritic cells. Blood.

[77] Kumar S. U., Sankar S., Younes S., Kumar D. T., Ahmad M.N., Okashah S.S., Kamaraj B., Al-Subaie A.M., Doss C. G.P., Zayed H. (2020). Deciphering the Role of Filamin B Calponin-Homology Domain in Causing the Larsen Syndrome, Boomerang Dysplasia, and Atelosteogenesis Type I Spectrum Disorders via a Computational Approach. Molecules.

[78] Zhao Y., Shapiro S.S., Eto M. (2016). F-actin clustering and cell dysmotility induced by the pathological W148R missense mutation of filamin B at the actin-binding domain. Am. J. Physiol.-Cell Physiol..

[79] Im Y.B., Jee M.K., Choi J.I., Cho H.T., Kwon O.H., Kang S.K. (2012). Molecular targeting of NOX4 for neuropathic pain after traumatic injury of the spinal cord. Cell Death Dis..

[80] Papadaki M., Rinotas V., Violitzi F., Thireou T., Panayotou G., Samiotaki M., Douni E. (2019). New Insights for RANKL as a Proinflammatory Modulator in Modeled Inflammatory Arthritis. Front. Immunol..

[81] Kim Y.S., Park H.J., Park J.H., Hong E.J., Jang G.-Y., Jung I.D., Han H.D., Lee S.-H., Vo M.-C., Lee J.-J. (2018). A novel function of API5 (apoptosis inhibitor 5), TLR4-dependent activation of antigen presenting cells. OncoImmunology.

[82] Li Z.-C., Xiao J., Peng J.-L., Chen J.-W., Ma T., Cheng G.-Q., Dong Y.-Q., Wang W., Liu Z.-D. (2014). Functional Annotation of Rheumatoid Arthritis and Osteoarthritis Associated Genes by Integrative Genome-Wide Gene Expression Profiling Analysis. PLoS ONE.

[83] Nejatbakhsh Samimi L., Farhadi E., Tahmasebi M.N., Jamshidi A., Sharafat Vaziri A., Mahmoudi M. (2020). NF-κB signaling in rheumatoid arthritis with focus on fibroblast-like synoviocytes. Autoimmun. Highlights.

[84] Lu M.-C., Lai N.-S., Chen H.-C., Yu H.-C., Huang K.-Y., Tung C.-H., Huang H.-B., Yu C.-L. (2013). Decreased microRNA(miR)-145 and increased miR-224 expression in T cells from patients with systemic lupus erythematosus involved in lupus immunopathogenesis. Clin. Exp. Immunol..

[85] Piatkov K.I., Brower C.S., Varshavsky A. (2012). The N-end rule pathway counteracts cell death by destroying proapoptotic protein fragments. Proc. Natl. Acad. Sci. USA.

[86] Piatkov K.I., Oh J.-H., Liu Y., Varshavsky A. (2014). Calpain-generated natural protein fragments as short-lived substrates of the N-end rule pathway. Proc. Natl. Acad. Sci. USA.

[87] Cypher L.R., Bielecki T.A., Adepegba O., Huang L., An W., Iseka F., Luan H., Tom E., Storck M.D., Hoppe A.D. (2016). CSF-1 receptor signalling is governed by pre-requisite EHD1 mediated receptor display on the macrophage cell surface. Cell. Signal..

[88] Mintz L., Galperin E., Pasmanik-Chor M., Tulzinsky S., Bromberg Y., Kozak C.A., Joyner A., Fein A., Horowitz M. (1999). EHD1—An EH-Domain-Containing Protein with a Specific Expression Pattern. Genomics.

[89] Miyauchi S., Maekawa T., Aoki Y., Miyazawa H., Tabeta K., Nakajima T., Yamazaki K. (2012). Oral infection with *Porphyromonas gingivalis* and systemic cytokine profile in C57BL/6.KOR-ApoE shl mice. J. Period. Res..

[90] Gordon E.M., Ravicz J.R., Liu S., Chawla S.P., Hall F.L. (2018). Cell cycle checkpoint control: The cyclin G1/Mdm2/p53 axis emerges as a strategic target for broad-spectrum cancer gene therapy-A review of molecular mechanisms for oncologists. Mol. Clin. Oncol..

[91] Inaba H., Kuboniwa M., Bainbridge B., Yilmaz Ö., Katz J., Shiverick K.T., Amano A., Lamont R.J. (2009). *Porphyromonas gingivalis* invades human trophoblasts and inhibits proliferation by inducing G1 arrest and apoptosis. Cell. Microbiol..

[92] Lin T., Ma Q., Zhang Y., Zhang H., Yan J., Gao C. (2018). MicroRNA-27a functions as an oncogene in human osteosarcoma by targeting CCNG1. Oncol. Lett..

[93] Velletri T., Huang Y., Wang Y., Li Q., Hu M., Xie N., Yang Q., Chen X., Chen Q., Shou P. (2021). Loss of p53 in mesenchymal stem cells promotes alteration of bone remodeling through negative regulation of osteoprotegerin. Cell Death Differ..

[94] Saito K., Murai J., Kajiho H., Kontani K., Kurosu H., Katada T. (2002). A Novel Binding Protein Composed of Homophilic Tetramer Exhibits Unique Properties for the Small GTPase Rab5. J. Biol. Chem..

[95] Basel-Vanagaite L., Sarig O., Hershkovitz D., Fuchs-Telem D., Rapaport D., Gat A., Isman G., Shirazi I., Shohat M., Enk C.D. (2009). RIN2 Deficiency Results in Macrocephaly, Alopecia, Cutis Laxa, and Scoliosis: MACS Syndrome. Am. J. Hum. Genet..

[96] Kato Y., Hagiwara M., Ishihara Y., Isoda R., Sugiura S., Komatsu T., Ishida N., Noguchi T., Matsushita K. (2014). TNF-α augmented *Porphyromonas gingivalis* invasion in human gingival epithelial cells through Rab5 and ICAM-1. BMC Microbiol..

[97] Hortle E., Tran L.V., Fontaine A.R., Pinello N., Wong J.J.-L., Britton W.J., Oehlers S.H. (2022). OXSR1 inhibits inflammasome activation by limiting potassium efflux during mycobacterial infection. Life Sci. Alliance.

[98] Hung C.-M., Peng C.-K., Yang S.-S., Shui H.-A., Huang K.-L. (2020). WNK4-SPAK modulates lipopolysaccharide-induced macrophage activation. Biochem. Pharmacol..

[99] Huang T., Zhou Y., Cao Y., Tao J., Zhou Z.-H., Hang D.-H. (2017). STK39, overexpressed in osteosarcoma, regulates osteosarcoma cell invasion and proliferation. Oncol. Lett..

