# Supplementary material for: Classifying Integrated Signature Molecules in Macrophages of Rheumatoid Arthritis, Osteoarthritis, and Periodontal Disease: An Omics-Based Study
Source: Curr Issues Mol Biol. 2022 Aug 6;44(8):3496–517. doi: 10.3390/cimb44080241 (PMC9406916; doi:10.3390/cimb44080241)
Supplement: Supplementary file 1 [file cimb-44-00241-s001.zip › cimb-1808574-supplementary.pdf]

**Table S1: Details of 72 Common DEGs**

| S.No. | Gene Name | | RA | | OA | | PG | |
|---|---|---|---|---|---|---|---|---|
| | *SYMBOL* | ENTREZID | P.Value | logFC | P.Value | logFC | P.Value | logFC |
| 1 | *ABHD5* | 51099 | 0.002443 | -0.56578 | 0.020179 | -3.10629 | 0.000509 | -0.70691 |
| 2 | *AKAP13* | 11214 | 2.15E-06 | 1.092525 | 0.01827 | 2.118771 | 0.0002 | 0.924739 |
| 3 | *API5* | 8539 | 5.17E-05 | 0.948967 | 0.044049 | -1.61151 | 0.003568 | -0.83848 |
| 4 | *ATE1* | 11101 | 7.50E-05 | -0.68299 | 0.010635 | -0.90888 | 0.002123 | -1.01828 |
| 5 | *ATP1B1* | 481 | 0.002511 | -1.45661 | 0.027328 | 3.118706 | 0.000517 | 0.776784 |
| 6 | *ATPAF1* | 64756 | 0.006199 | -0.65636 | 0.00396 | -1.31407 | 0.002152 | -0.89802 |
| 7 | *B4GALT5* | 9334 | 0.010165 | 0.746297 | 0.040347 | 2.219408 | 2.15E-05 | 1.346279 |
| 8 | *BLOC1S2* | 282991 | 1.10E-06 | -1.19876 | 0.017607 | -0.66175 | 0.000387 | -0.6478 |
| 9 | *CAB39L* | 81617 | 3.15E-07 | -1.92443 | 0.003059 | 4.237594 | 0.000456 | -1.23121 |
| 10 | *CCDC66* | 285331 | 0.001966 | -0.64955 | 0.003516 | -3.63235 | 0.000159 | -0.9599 |
| 11 | *CCNG1* | 900 | 0.000262 | -0.7122 | 0.008933 | -1.613 | 0.001181 | -0.78366 |
| 12 | *CD300A* | 11314 | 0.011903 | 0.69621 | 0.010745 | 3.115973 | 5.86E-05 | -0.74556 |
| 13 | *CHST13* | 166012 | 2.67E-06 | -2.44549 | 0.034288 | 0.504662 | 0.002919 | -0.70979 |
| 14 | *CISD1* | 55847 | 0.00416 | -1.03413 | 0.031482 | -2.78761 | 0.002289 | -0.80914 |
| 15 | *CLIP4* | 79745 | 0.002999 | -0.64836 | 0.011162 | -3.54783 | 0.000257 | -1.12684 |
| 16 | *DAGLB* | 221955 | 0.001786 | 1.117383 | 0.006518 | 1.843946 | 0.000164 | -0.76796 |
| 17 | *DNAAF1* | 123872 | 0.006008 | 1.918584 | 0.01782 | 4.193941 | 2.57E-06 | 2.29468 |
| 18 | *EHD1* | 10938 | 1.31E-05 | 2.130844 | 0.041034 | -2.59141 | 4.34E-06 | 2.083501 |
| 19 | *ERCC1* | 2067 | 0.000607 | 0.919528 | 0.015691 | -2.8963 | 0.000494 | -0.64705 |
| 20 | *ETFA* | 2108 | 5.67E-05 | -0.62781 | 0.014883 | 1.389631 | 0.002729 | -0.52794 |
| 21 | *FBXO28* | 23219 | 0.002614 | -0.52079 | 0.008138 | 0.525873 | 0.001003 | -0.77731 |
| 22 | *FH* | 2271 | 0.000411 | -0.6469 | 0.007016 | -3.76123 | 0.001566 | -1.09595 |
| 23 | *FMNL1* | 752 | 5.56E-08 | 1.528973 | 0.026812 | -2.89386 | 3.80E-05 | 1.036113 |
| 24 | *FSD1L* | 83856 | 0.000751 | 1.108685 | 0.015012 | 3.816089 | 0.001085 | 1.099068 |
| 25 | *GFM2* | 84340 | 0.000152 | -1.04212 | 0.008164 | -2.76709 | 0.000103 | -0.89154 |
| 26 | *GGCT* | 79017 | 1.70E-05 | -0.8628 | 0.012418 | 2.605323 | 0.000214 | -0.75831 |
| 27 | *GZF1* | 64412 | 0.007052 | 0.947051 | 0.001678 | -3.57112 | 0.000761 | -1.47368 |

| | | | | | | | | |
|---|---|---|---|---|---|---|---|---|
| 28 | *HNMT* | 3176 | 1.09E-05 | -1.1326 | 0.007327 | -1.42762 | 0.001013 | -0.82189 |
| 29 | *IER3* | 8870 | 1.79E-05 | 1.58268 | 0.036425 | 1.61056 | 8.52E-09 | 3.691343 |
| 30 | *IL6* | 3569 | 0.005851 | 1.426971 | 0.04803 | -2.54602 | 1.02E-08 | 6.170007 |
| 31 | *INTS10* | 55174 | 0.012768 | -0.81647 | 0.023895 | 2.882221 | 0.000466 | -0.83698 |
| 32 | *KBTBD7* | 84078 | 2.97E-09 | -3.02472 | 0.041311 | 1.668666 | 0.001699 | -0.89937 |
| 33 | *KDM7A* | 80853 | 2.69E-05 | 1.081884 | 0.047432 | 2.1846 | 0.002626 | 0.819238 |
| 34 | *KLHL28* | 54813 | 6.93E-05 | -0.85664 | 0.034672 | 3.240225 | 8.10E-07 | 1.468859 |
| 35 | *LRP12* | 29967 | 0.007109 | -1.14802 | 0.020469 | 0.644235 | 0.000774 | 1.10113 |
| 36 | *MBLAC2* | 153364 | 9.60E-05 | -1.51133 | 0.000336 | -3.85063 | 1.97E-05 | -0.92086 |
| 37 | *MBTPS2* | 51360 | 0.000197 | -0.60447 | 1.19E-05 | -1.72224 | 0.003269 | -0.7793 |
| 38 | *MCEE* | 84693 | 7.85E-05 | -0.73856 | 0.02115 | 0.793102 | 0.000452 | -1.11363 |
| 39 | *METTL25* | 84190 | 0.001244 | -0.63086 | 0.037937 | 0.724949 | 0.003103 | -0.70809 |
| 40 | *MLLT10* | 8028 | 0.001097 | -0.57958 | 0.047278 | 2.742738 | 4.65E-05 | -0.99596 |
| 41 | *MOSPD1* | 56180 | 5.56E-09 | -1.47123 | 0.003234 | 3.405434 | 0.003576 | -0.94579 |
| 42 | *MPP5* | 64398 | 3.46E-08 | -2.4948 | 0.030964 | 2.322848 | 0.003249 | -0.8469 |
| 43 | *MRPL39* | 54148 | 0.009186 | 0.88486 | 0.035387 | 2.047736 | 0.000249 | -0.78253 |
| 44 | *MTIF3* | 219402 | 0.001492 | -0.78276 | 0.045675 | -0.6177 | 4.02E-05 | -1.21492 |
| 45 | *NLRP3* | 114548 | 1.57E-07 | 4.303286 | 0.016642 | -1.11036 | 2.44E-05 | 2.918478 |
| 46 | *PMEPA1* | 56937 | 0.000682 | 1.412264 | 0.041631 | -2.23103 | 0.000562 | 0.656428 |
| 47 | *PNKD* | 25953 | 1.72E-05 | -1.16772 | 0.006833 | -0.73991 | 0.000426 | -0.82808 |
| 48 | *PNRC1* | 10957 | 0.007087 | 0.597413 | 0.041306 | 1.757568 | 1.50E-06 | 1.880961 |
| 49 | *PPP1R18* | 170954 | 0.001211 | 0.597533 | 0.047425 | 1.476029 | 7.71E-06 | 1.106381 |
| 50 | *PSTPIP1* | 9051 | 1.46E-06 | 1.878514 | 0.045162 | 1.495317 | 0.000169 | -0.81923 |
| 51 | *RCBTB2* | 1102 | 0.000346 | -0.78763 | 0.048169 | 1.972613 | 0.001071 | -1.38603 |
| 52 | *RFK* | 55312 | 5.99E-05 | 1.03999 | 0.018353 | 2.625587 | 0.001629 | -0.76247 |
| 53 | *RGS16* | 6004 | 0.005979 | 1.14111 | 0.022101 | 2.358466 | 7.23E-06 | 1.065291 |
| 54 | *RHNO1* | 83695 | 0.000957 | -0.59207 | 0.030542 | 2.126232 | 0.001321 | -1.00823 |
| 55 | *RIN2* | 54453 | 7.06E-05 | 3.051098 | 0.042093 | -1.46963 | 0.000764 | -1.55874 |
| 56 | *RPAP3* | 79657 | 0.002535 | -0.53975 | 0.011583 | -0.78553 | 0.000388 | -1.59776 |
| 57 | *RPGR* | 6103 | 0.002118 | 0.651429 | 0.043931 | 2.703224 | 0.00043 | 0.72406 |
| 58 | *RPUSD3* | 285367 | 4.03E-05 | -1.10226 | 0.046301 | -0.58511 | 0.000135 | -1.08247 |

| | | | | | | | | |
|---|---|---|---|---|---|---|---|---|
| 59 | *SAMHD1* | 25939 | 0.000158 | 0.653058 | 0.016753 | -3.903 | 0.003645 | -1.28668 |
| 60 | *SAR1B* | 51128 | 1.06E-05 | -0.73891 | 0.000472 | 0.842645 | 0.000388 | -0.78643 |
| 61 | *SLC1A2* | 6506 | 0.011151 | 1.88593 | 0.022717 | -1.38018 | 9.13E-07 | 2.859791 |
| 62 | *SLC20A1* | 6574 | 0.000207 | 0.571868 | 0.013494 | -1.41614 | 0.001468 | -0.77487 |
| 63 | *SQSTM1* | 8878 | 2.40E-06 | -1.3107 | 0.030014 | -2.45249 | 0.002504 | 0.90745 |
| 64 | *SYNRG* | 11276 | 0.004741 | -0.59859 | 0.007395 | -2.74597 | 0.000155 | -0.76539 |
| 65 | *TAPBP* | 6892 | 3.92E-05 | 0.913076 | 0.030057 | 2.118014 | 0.000712 | 0.909168 |
| 66 | *TBC1D12* | 23232 | 0.001189 | -0.73789 | 0.003551 | 0.655343 | 0.001446 | 0.546048 |
| 67 | *THEM4* | 117145 | 0.000146 | 0.778283 | 0.039497 | 2.014082 | 0.00089 | -0.7336 |
| 68 | *TMCC1* | 23023 | 1.23E-07 | -1.21068 | 6.16E-06 | 5.415481 | 0.0004 | -1.14181 |
| 69 | *TMED8* | 283578 | 2.75E-05 | -0.8267 | 0.021837 | 2.55728 | 0.003496 | -0.83207 |
| 70 | *TMEM263* | 90488 | 0.000919 | -0.73695 | 0.034029 | -1.29565 | 0.003673 | -0.90391 |
| 71 | *TNF* | 7124 | 2.39E-06 | 2.421162 | 0.011977 | -3.41913 | 5.54E-08 | 5.240661 |
| 72 | *TRAF3* | 7187 | 3.54E-06 | -1.18885 | 0.03868 | -0.81485 | 0.001364 | 0.6176 |

**Table S2: GeneMANIA Output for Protein-Protein interaction network**

| **Gene name** | **Node type** | **Name** | **Synonym** | **Log score** | **Score** | **RefSeq mRNA ID** | **RefSeq Protein ID** | **Uniprot ID** | **Ensembl Gene ID** | **Ensembl Protein ID** | **Entrez Gene ID** |
|---|---|---|---|---|---|---|---|---|---|---|---|
| RPAP3 | query | H__sapiens__4_-701058 | spag | -0.31775 | 0.727781 | NM_024604 | NP_078880 | Q9H6T3 | ENSG00000005175 | ENSP00000401823 | 79657 |
| ABHD5 | query | H__sapiens__4_-701259 | NCIE2 | -0.26487 | 0.767309 | NM_016006 | NP_057090 | Q8WTS1 | ENSG00000011198 | ENSP00000412014 | 51099 |
| MBTPS2 | query | H__sapiens__4_-701288 | S2P | -0.26912 | 0.764052 | NM_015884 | NP_056968 | O43462 | ENSG00000012174 | ENSP00000368798 | 51360 |
| TXNL1 | result | H__sapiens__4_-702896 | TXNL | -4.30402 | 0.013514 | NM_004786 | NP_004777 | O43396 | ENSG00000091164 | ENSP00000468165 | 9352 |

| | | | | | | | | | | | |
|---|---|---|---|---|---|---|---|---|---|---|---|
| FH | query | H__sapiens__4_-702904 | fumarase | -0.45588 | 0.633892 | NM_000143 | NP_000134 | P07954 | ENSG00000091483 | ENSP00000355518 | 2271 |
| PSMD10 | result | H__sapiens__4_-703554 | | -3.96941 | 0.018885 | NM_170750 | NP_736606 | O75832 | ENSG00000101843 | ENSP00000361370 | 5716 |
| CAB39L | query | H__sapiens__4_-703662 | MO2L | -0.1529 | 0.858213 | NM_030925 | NP_112187 | Q9H9S4 | ENSG00000102547 | ENSP00000479669 | 81617 |
| NUBP1 | result | H__sapiens__4_-703774 | NBP35 | -4.28925 | 0.013715 | NM_002484 | NP_002475 | P53384 | ENSG00000103274 | ENSP00000461746 | 4682 |
| ATE1 | query | H__sapiens__4_-704415 | | -0.1426 | 0.867104 | NM_007041 | NP_008972 | O95260 | ENSG00000107669 | ENSP00000444852 | 11101 |
| XPNPEP1 | result | H__sapiens__4_-704464 | XPNPEPL1 | -4.39697 | 0.012315 | NM_020383 | NP_065116 | Q9NQW7 | ENSG00000108039 | ENSP00000421566 | 7511 |
| TBC1D12 | query | H__sapiens__4_-704479 | KIAA0608 | -0.21905 | 0.803279 | NM_015188 | NP_056003 | O60347 | ENSG00000108239 | ENSP00000225235 | 23232 |
| DRG2 | result | H__sapiens__4_-704537 | | -3.50461 | 0.030058 | NM_001388 | NP_001379 | P55039 | ENSG00000108591 | ENSP00000464244 | 1819 |
| EHD1 | query | H__sapiens__4_-704713 | PAST1 | -0.41375 | 0.661163 | NM_006795 | NP_006786 | Q9H4M9 | ENSG00000110047 | ENSP00000479153 | 10938 |
| PPP6R3 | result | H__sapiens__4_-704720 | SAPS3 | -3.79507 | 0.022481 | NM_018312 | NP_060782 | Q5H9R7 | ENSG00000110075 | ENSP00000437329 | 55291 |
| PANX1 | result | H__sapiens__4_-704737 | UNQ2529 | -4.4176 | 0.012063 | NM_015368 | NP_056183 | Q96RD7 | ENSG00000110218 | ENSP00000411461 | 24145 |
| TAPBP | query | H__sapiens__4_-705052 | TAPA | -0.26741 | 0.765358 | NM_172209 | NP_757346 | O15533 | ENSG00000236490 | ENSP00000450358 | 6892 |
| CCNG1 | query | H__sapiens__4_-705154 | CCNG | -0.39533 | 0.673455 | NM_199246 | NP_954854 | P51959 | ENSG00000113328 | ENSP00000427086 | 900 |

| | | | | | | | | | | | |
|---|---|---|---|---|---|---|---|---|---|---|---|
| CLIP4 | query | H__sapiens__4_-705386 | RSNL2 | -0.22142 | 0.80138 | NM_024692 | NP_078968 | Q8N3C7 | ENSG00000115295 | ENSP00000414417 | 79745 |
| MTIF3 | query | H__sapiens__4_-706211 | IF3(mt) | -0.10382 | 0.901384 | NM_152912 | NP_690876 | Q9H2K0 | ENSG00000122033 | ENSP00000384659 | 219402 |
| CISD1 | query | H__sapiens__4_-706306 | ZCD1 | -0.26017 | 0.770921 | NM_018464 | NP_060934 | Q9NZ45 | ENSG00000122873 | ENSP00000363041 | 55847 |
| ATPAF1 | query | H__sapiens__4_-706375 | FLJ22351 | -0.14798 | 0.862448 | NM_022745 | NP_073582 | Q5TC12 | ENSG00000123472 | ENSP00000460964 | 64756 |
| PMEPA1 | query | H__sapiens__4_-706474 | TMEPAI | -0.21324 | 0.807959 | NM_199171 | NP_954640 | Q969W9 | ENSG00000124225 | ENSP00000401506 | 56937 |
| MTRR | result | H__sapiens__4_-706488 | cblE | -4.44617 | 0.011723 | NM_024010 | NP_076915 | Q9UBK8 | ENSG00000124275 | ENSP00000427416 | 4552 |
| GTF3C4 | result | H__sapiens__4_-706642 | TFIIIC90 | -4.43788 | 0.011821 | NM_012204 | NP_036336 | Q9UKN8 | ENSG00000125484 | ENSP00000431378 | 9329 |
| KTN1 | result | H__sapiens__4_-706837 | KNT | -4.23298 | 0.014509 | NM_004986 | NP_004977 | Q86UP2 | ENSG00000126777 | ENSP00000452576 | 3895 |
| METTL25 | query | H__sapiens__4_-706931 | FLJ22789 | -0.11605 | 0.890429 | NM_032230 | NP_115606 | Q8N6Q8 | ENSG00000127720 | ENSP00000450317 | 84190 |
| ATP6V1E1 | result | H__sapiens__4_-707388 | ATP6V1E | -4.21286 | 0.014804 | NM_001696 | NP_001687 | P36543 | ENSG00000131100 | ENSP00000398932 | 529 |
| RIN2 | query | H__sapiens__4_-707618 | | -0.23488 | 0.790665 | NM_018993 | NP_061866 | Q8WYP3 | ENSG00000132669 | ENSP00000391239 | 54453 |
| FLNB | result | H__sapiens__4_-708196 | TABP | -3.99978 | 0.01832 | NM_001457 | NP_001448 | O75369 | ENSG00000136068 | ENSP00000420213 | 2317 |
| IER3 | query | H__sapiens__4_-708443 | IEX-1L | -0.36221 | 0.696133 | NM_003897 | NP_003888 | P46695 | ENSG00000237155 | ENSP00000412861 | 8870 |

| | | | | | | | | | | | |
|---|---|---|---|---|---|---|---|---|---|---|---|
| PSTPIP1 | query | H__sapiens__4_-708945 | PSTPIP | -0.4135 | 0.661331 | NM_003978 | NP_003969 | O43586 | ENSG00000140368 | ENSP00000454127 | 9051 |
| TPM1 | result | H__sapiens__4_-708957 | CMH3 | -4.0786 | 0.016931 | NM_001301289 | NP_001288218 | P09493 | ENSG00000140416 | ENSP00000477929 | 7168 |
| ATP1B1 | query | H__sapiens__4_-709316 | ATP1B | -0.28269 | 0.753752 | NM_001677 | NP_001668 | P05026 | ENSG00000143153 | ENSP00000477015 | 481 |
| RGS16 | query | H__sapiens__4_-709359 | RGS-r | -0.24755 | 0.780714 | NM_002928 | NP_002919 | O15492 | ENSG00000143333 | ENSP00000356529 | 6004 |
| SLC20A1 | query | H__sapiens__4_-709520 | PiT-1 | -0.29409 | 0.745208 | NM_005415 | NP_005406 | Q8WUM9 | ENSG00000144136 | ENSP00000413393 | 6574 |
| PNRC1 | query | H__sapiens__4_-709821 | PROL2 | -0.35814 | 0.698979 | NM_006813 | NP_006804 | Q12796 | ENSG00000146278 | ENSP00000358484 | 10957 |
| HNMT | query | H__sapiens__4_-710303 | | -0.25528 | 0.774702 | NM_006895 | NP_008826 | P50135 | ENSG00000150540 | ENSP00000419415 | 3176 |
| BAG3 | result | H__sapiens__4_-710472 | | -3.66564 | 0.025588 | NM_004281 | NP_004272 | O95817 | ENSG00000151929 | ENSP00000410036 | 9531 |
| SAR1B | query | H__sapiens__4_-710569 | SARA2 | -0.34448 | 0.708589 | NM_016103 | NP_057187 | Q9Y6B6 | ENSG00000152700 | ENSP00000426335 | 51128 |
| SQSTM1 | query | H__sapiens__4_-711566 | PDB3 | -0.40068 | 0.669861 | NM_003900 | NP_003891 | Q13501 | ENSG00000161011 | ENSP00000487071 | 8878 |
| NLRP3 | query | H__sapiens__4_-711803 | PYPAF1 | -0.3204 | 0.725859 | NM_183395 | NP_899632 | Q96P20 | ENSG00000162711 | ENSP00000375704 | 114548 |
| PGGT1B | result | H__sapiens__4_-712287 | GGTI | -4.19668 | 0.015045 | NM_005023 | NP_005014 | P53609 | ENSG00000164219 | ENSP00000404676 | 5229 |
| API5 | query | H__sapiens__4_-712781 | API5L1 | -0.38803 | 0.678392 | NM_006595 | NP_006586 | Q9BZZ5 | ENSG00000166181 | ENSP00000436436 | 8539 |

| | | | | | | | | | | | |
|---|---|---|---|---|---|---|---|---|---|---|---|
| STAT6 | resu lt | H__sapiens__4_-712964 | IL-4-STAT | -3.66551 | 0.025591 | NM_003153 | NP_003144 | P42226 | ENSG00000166888 | ENSP00000452394 | 6778 |
| AKAP13 | quer y | H__sapiens__4_-713896 | PROTO-LB | -0.22662 | 0.797222 | NM_007200 | NP_009131 | Q12802 | ENSG00000170776 | ENSP00000483091 | 11214 |
| RHNO1 | quer y | H__sapiens__4_-714145 | RHINO | -0.14504 | 0.864984 | NM_001257098 | NP_001244027 | Q9BSD3 | ENSG00000171792 | ENSP00000479598 | 83695 |
| OXSR1 | resu lt | H__sapiens__4_-714418 | KIAA1101 | -4.28476 | 0.013777 | NM_005109 | NP_005100 | O95747 | ENSG00000172939 | ENSP00000415851 | 9943 |
| FMNL1 | quer y | H__sapiens__4_-716857 | FMNL | -0.31664 | 0.728595 | NM_005892 | NP_005883 | O95466 | ENSG00000184922 | ENSP00000468301 | 752 |
| BLOC1S2 | quer y | H__sapiens__4_-717993 | MGC10120 | -0.18041 | 0.834931 | NM_173809 | NP_776170 | Q6QNY1 | ENSG00000196072 | ENSP00000484340 | 282991 |
| PSMD12 | resu lt | H__sapiens__4_-718364 | Rpn5 | -4.30802 | 0.01346 | NM_174871 | NP_777360 | O00232 | ENSG00000197170 | ENSP00000463017 | 5718 |
| STK39 | resu lt | H__sapiens__4_-718830 | SPAK | -3.7356 | 0.023859 | NM_013233 | NP_037365 | Q9UEW8 | ENSG00000198648 | ENSP00000348278 | 27347 |
| TXNIP | resu lt | H__sapiens__4_-751999 | VDUP1 | -4.36564 | 0.012707 | NM_006472 | NP_006463 | Q9H3M7 | ENSG00000265972 | ENSP00000462521 | 10628 |
| SYNRG | quer y | H__sapiens__4_-756501 | SYNG | -0.20444 | 0.815106 | NM_198882 | NP_942583 | Q9UMZ2 | ENSG00000275066 | ENSP00000488863 | 11276 |
